# Carbon Catabolite Repression and the Related Genes of *ccpA*, *ptsH* and *hprK* in *Thermoanaerobacterium aotearoense*

**DOI:** 10.1371/journal.pone.0142121

**Published:** 2015-11-05

**Authors:** Muzi Zhu, Yanping Lu, Jufang Wang, Shuang Li, Xiaoning Wang

**Affiliations:** 1 Provincial Key Laboratory of Fermentation and Enzyme Engineering, School of Bioscience and Bioengineering, South China University of Technology, Guangzhou, China; 2 State Key Laboratory of Kidney, the Institute of Life Sciences, Chinese PLA General Hospital, Beijing, China; LSU Health Sciences Center School of Dentistry, UNITED STATES

## Abstract

The strictly anaerobic, Gram-positive bacterium, *Thermoanaerobacterium aotearoense* SCUT27, is capable of producing ethanol, hydrogen and lactic acid by directly fermenting glucan, xylan and various lignocellulosically derived sugars. By using non-metabolizable and metabolizable sugars as substrates, we found that cellobiose, galactose, arabinose and starch utilization was strongly inhibited by the existence of 2-deoxyglucose (2-DG). However, the xylose and mannose consumptions were not markedly affected by 2-DG at the concentration of one-tenth of the metabolizable sugar. Accordingly, *T*. *aotearoense* SCUT27 could consume xylose and mannose in the presence of glucose. The carbon catabolite repression (CCR) related genes, *ccpA*, *ptsH* and *hprK* were confirmed to exist in *T*. *aotearoense* SCUT27 through gene cloning and protein characterization. The highly purified Histidine-containing Protein (HPr) could be specifically phosphorylated at Serine 46 by HPr kinase/phosphatase (HPrK/P) with no need to add fructose-1,6-bisphosphate (FBP) or glucose-6-phosphate (Glc-6-P) in the reaction mixture. The specific protein-interaction of catabolite control protein A (CcpA) and phosphorylated HPr was proved via affinity chromatography in the absence of formaldehyde. The equilibrium binding constant (*K*_D_) of CcpA and HPrSerP was determined as 2.22 ± 0.36 nM by surface plasmon resonance (SPR) analysis, indicating the high affinity between these two proteins.

## Introduction

In nature, microorganisms develop a mechanism to metabolize a variety of carbon sources to acquire energy in order to survive complex environment. Most bacteria prefer to utilize the readily accessible sugars and yield a maximum profit in the competition with other microorganisms [1]. The phenomenon that some sugars are predominately utilized is usually determined by the carbon catabolite regulation (CCR) mechanism [2, 3]. For many bacteria, glucose is the most preferred source of carbon. Its existence usually induces the suppression of genes or operons that encode metabolites are required for the secondary carbon source metabolism. Thus, the regulation mechanism is also referred as carbon catabolite repression (also abbreviated as CCR) [4–6].

CCR of gene expression is a universal phenomenon found in almost all living microorganisms [7]. However, the mechanism varies depending on the organisms and the combined activities of global and operon-specific regulatory genes [4, 8–10]. In low G+C gram-positive bacteria, such as Firmicutes including *Clostridia* and *Bacilli*, the Histidine-containing Protein (HPr), bifunctional HPr kinase/phosphatase (HPrK/P) and catabolite control protein A (CcpA) are involved in the CCR regulating mechanism [4, 8, 10, 11]. The phosphorylation of HPr at different spots catalyzed by HPrK/P controls the CCR-related gene transcription and expression [12]. CcpA is a DNA binding protein, and a member of the LacI/GalR family of transcriptional repressors/activators [13]. As response to the presence of glucose or other preferable carbon sources in the growth medium, HPr is phosphorylated at the Ser-46 residue by ATP-dependent HPrK/P to form seryl-phosphorylated HPr (HPrSerP) [4, 10, 14]. CcpA binds to a consensus semi-palindromic DNA sequence in the control region of a target gene, a catabolite-responsive element (*cre*) when it interacts with HPrSerP to form a complex. As a consequence, gene transcription is activated or repressed [4, 12].

*Thermoanaerobacterium* sp. is one type of the so-called thermophilic, Gram-positive, anaerobic bacteria (TGPAs) [15], which are able to utilize a variety of carbohydrates as carbon sources, including many hexoses, pentoses, cellobiose, dextran and xylan, to support cell growth [16–19]. The anaerobic and thermophilic characteristics render the strain an attractive bacterium for biofuel production from renewable sources. Shaw *et al*. redirected the carbon flux of *Thermoanaerobacterium saccharolyticum* almost exclusively to ethanol. Expression of heterologous urease genes in *T*. *saccharolyticum* resulted in the production of 54 g/L ethanol, which was one of the highest titers reported for *Thermoanaerobacterium* [20]. We also reported that the changes of total cellular NADH distribution resulted in a remarkable increase of hydrogen production by *Thermoanaerobacterium aotearoense* [16]. Although these strains can co-utilize many of the sugars derived from lignocellulosic biomass for cellulosic energy production, they exhibit carbon source preferences when cultured in sugar mixture. Studies on the related strain *Thermoanaerobacter* sp. X514 showed that it can efficiently metabolize hexose and pentose in parallel, indicating an absence of CCR [15]. Furthermore, the *Thermoanaerobacter* glycobiome revealed the dynamics and the cooperating nature of pentose and hexose co-utilization, which were obviously regulated by transcriptional antiterminators of the BglG family [15]. However, the similar firmicute strain, *T*. *saccharolyticum* M2476, was reported to suffer the cAMP-independent CCR mechanism [5].

In this study, we firstly investigated the CCR of lignocellulosic biomass derived sugars in *T*. *aotearoense* SCUT27 using glucose analogue, 2-deoxyglucose (2-DG) as inhibitor. Then the CCR-related genes and proteins in this strain were sequenced and functionally characterized.

## Materials and Methods

### Strains, plasmids and culture conditions

The bacteria and plasmids used in this research are listed in Table 1. *T*. *aotearoense* SCUT27 (CGMCC NO.10833) was identified and preserved in our group as described in [16]. Cell culture of *T*. *aotearoense* was conducted in 125-mL serum bottles containing 50 mL modified MTC medium at 55°C with a nitrogen gas headspace in the pressure of 0.14 × 10^6^ Pa. The *ccpA*-deficient *B*. *subtilis* MA-1 was kindly provided by Prof. S. Yang [6]. All the strains of *B*. *subtilis* and *Escherichia coli* (*E*. *coli*) were cultivated aerobically at 37°C in Luria-Bertani (LB) medium supplemented with appropriate antibiotics. When necessary, 5 μg/mL of chloramphenicol, 50 μg/mL of kanamycin, 100 μg/mL of ampicillin, or 100 μg/mL of spectinomycin was added into the medium.

**Table 1:** Strains and plasmids used in this study.

Strains or plasmids	Description	Source or reference
*T*. *aotearoense* SCUT27	*Δpta*, *Δack*	Constructed and incubated in our lab.
*B*. *subtilis* 168	*trpC2*	Guangdong Culture Collection Center of Microbiology, China.
*B*. *subtilis* MA-1	168 derivate, *trpC2*, *ccpA*::*spec* ^*r*^	Offered by Prof. S. Yang (Institute of Plant Physiology and Ecology, Shanghai)[6]
*B*. *subtilis* TA-1	168 derivate, *trpC2*, *ccpA*::*spec* ^*r*^/pHT01-T*ccpA*	This study
*E*. *coli* DH5α		Invitrogen, CA, USA
**Plasmids**	**Description**	**Source**
pMD^®^18-T	*Amp* ^*r*^, *E*. *coli* cloning vector	TaKaRa, Dalian, China
pMD-T*ccpA*	pMD18-T derivate, *Amp* ^*r*^, with *ccpA* from *T*. *aotearoense*	This study
pMD-T*hpr*	pMD18-T derivate, *Amp* ^*r*^, with *hpr* from *T*. *aotearoense*	This study
pMD-T*hprK*	pMD18-T derivate, *Amp* ^*r*^, with *hprK* from *T*. *aotearoense*	This study
pHT01	*Amp* ^*r*^, *Cm* ^*r*^, *E*. *coli—B*. *stubtilis* shuttle vector	MoBiTec, Göttingen, Germany
pHT01-T*ccpA*	Derived from pHT01 containing the T-*ccpA* gene	This study
pET30a	*Kan* ^*r*^, *E*. *coli* expression vector	Novagen, Wisconsin, USA
pET-CcpA	pET30a derivate, *Kan* ^*r*^, with *ccpA* from *T*. *aotearoense* and a C-terminal His-tag	This study
pET-CcpA-NH	pET30a derivate, *Kan* ^*r*^, with *ccpA* from *T*. *aotearoense*	This study
pET-HPr	pET30a derivate, *Kan* ^*r*^, with *hpr* from *T*. *aotearoense* and a C-terminal His-tag	This study
pET-HPrM	pET-HPr derivate, the Ser46 in HPr was mutated to Ala, *Kan* ^*r*^	This study
pET-HPrK	pET30a derivate, *Kan* ^*r*^, with *hprK* from *T*. *aotearoense* and a C-terminal His-tag	This study

### 2-DG repression assays

2-DG-induced CCR repression was assayed in anaerobic culture tubes containing 10 mL liquid MTC media. Overnight culture was inoculated in fresh media containing 5 g/L of different carbon sources to an OD_600_ of 0.15 in the absence or presence of 2-DG. When xylose was used as carbon source, 2-DG was added to a final concentration of 0.005, 0.05, 0.5 or 5 g/L respectively. To determine the other carbon source utilization, cells were cultured with 0.5 g/L of 2-DG. Cell growth was monitored by measuring OD_600_ using Genesys^®^ 10vis spectrometer (Thermo Scientific, Waltham, MA).

### Glucose repression assays

To confirm whether CCR phenomenon exits in *T*. *aotearoense* SCUT27, cells were cultured in anaerobic serum bottles at 55°C containing 50 mL MTC medium using 5 g/L glucose and 5 g/L a different carbohydrate as carbon source, *i*.*e*. xylose, mannose, galactose, arabinose and cellobiose. Samples were taken at every 3 h to detect the residual sugar concentration by high performance liquid chromatography (HPLC, Waters 2695, Milford, MA) equipped with an Aminex 87H column (Bio-Rad Laboratories Inc., Hercules, CA, USA) and a refractive index detector (Waters 2414, Milford, MA). The column temperature was set at 60°C, and the detector temperature was 35°C. The mobile phase was 5 mM H_2_SO_4_ at a flow rate of 0.6 mL/min. All samples were passed through 0.22-μm filters before loading.

### Gene cloning and sequence analysis

Genomic DNA of *T*. *aotearoense* SCUT27 was prepared using a genome extraction kit (Sangon, Shanghai, China). The CcpA gene (*ccpA*), HPr gene (*ptsH*) and HPrK/P gene (*hprK*) were amplified by PCR reaction using TaKaRa Ex Tag Polymerase (TaKaRa, Dalian, China) with the genomic DNA of *T*. *aotearoense* SCUT27 as the template. The corresponding primer pairs were listed in Table 2. The amplified *ccpA*, *ptsH* and *hprK* were ligated with pMD^®^18-T to yield pMD-T*ccpA*, pMD-T*ptsH* and pMD-T*hprK*, separately. DNA sequencing was performed by Sangon Biotech company (Shanghai, China).

**Table 2:** Primers used in this study.

Primers	Sequence (from 5' to 3' ends) ^a^	Description
*ccpA*-F	TCGTTACGCGTGTGAATGCTACWATTAAAGATGT	Forward primer for the full-length of *ccpA*.
*ccpA*-R	TGTGTGACGTCCTATTTTTTTTCTTCACCATATCCT	Reverse primer for the full-length of *ccpA*.
Tc-F	GAGCGACATATGAATGCTACAATTAAAGATGT	Forward primer for the full-length of *ccpA*, expressed in *E*. *coli* with a C-terminal His-tag.
Tc-R1	TTTACCCTCGAGTTTTTTTTCTTCACCATATCCT	Reverse primer for the full-length of *ccpA*, expressed in *E*. *coli* with a C-terminal His-tag.
Tc-R2	TTTACCGGATCCCTATTTTTTTTCTTCACCATATC	Reverse primer for the full-length of *ccpA*, expressed in *E*. *coli* without a His-tag.
*hpr*-F	GAGCGACATATGACTGAAAAGACAGTAGAAAT	Forward primer for the full-length of *hpr*, expressed in *E*. *coli* with a C-terminal His-tag.
*hpr*-R	GTTACCAAGCTTTTCTTCTCCAAATTTTGAATCAAT	Reverse primer for the full-length of *hpr*, expressed in *E*. *coli* with a C-terminal His-tag.
*hpr*-MR	CATAATACCCATGAT**CGC**TTTCGCA	Overlapping PCR primers for the mutation of Ser46-Ala in HPr, the mutation sites were in bold.
*hpr*-MF	TGCGAAA**GCG**ATCATGGGTATTATG	Overlapping PCR primers for the mutation of Ser46-Ala in HPr, the mutation sites were in bold.
*hprK*-F	GAGCGACATATGTTRAGTGTNTCWGTTGMGGATT	Forward primer for the full-length of *hprK*, expressed in *E*. *coli* with a C-terminal His-tag.
*hprK*-R	GTTACCAAGCTTWGTKGAAATYTGTTTCAACAAT	Reverse primer for the full-length of *hprK*, expressed in *E*. *coli* with a C-terminal His-tag.
16s-qF	CTGCCTGTAAGACTGGGATAAC	Forward primer for 16s-rRNA in RT-PCR.
16s-qR	CATCTGTAAGTGGTAGCCGAAG	Reverse primer for 16s-rRNA in RT-PCR.
*amyE*-qF	TCGCGGGAGTGCTTTATTT	Forward primer for *amyE* in RT-PCR.
*amyE*-qR	TTTCCATTCGGGTTCGAGAG	Reverse primer for *amyE* in RT-PCR.
*xylA*-qF	CGACCACCCATCAATACGATAC	Forward primer for *xylA* in RT-PCR
*xylA*-qR	GGCATGATTGGCTTCAAGATTT	Reverse primer for *xylA* in RT-PCR
*xylB*-qF	GGAGCCAATGGACTGCTATAC	Forward primer for *xylB* in RT-PCR
*xylB*-qR	GCTCCATCCATTCCGATCAA	Reverse primer for *xylB* in RT-PCR

^a^ Underlined nucleotides indicate the restriction enzyme sites.

Sequence analysis was carried out manually using the BLAST algorithm with all nucleotides deposited in the GenBank database (http://blast.ncbi.nlm.nih.gov/). The multiple sequence alignment tool Clustal X [21] was used for multiple protein sequence alignment. For phylogenetic analyses, phylogenetic relationships were inferred using the Neighbor-Joining (NJ) method by MEGA 4.0 [22], and the results were evaluated by bootstrap resampling with 1000 replicates. The accession numbers of reference strains used in polygenetic tree mapping were listed in related figure.

### Functional analysis of CcpA in *B*. *subtilis*

#### Introduction of cloned CcpA into the *ccpA*-deficient *B*. *subtilis* MA-1

To verify the function of the putative CcpA from *T*. *aotearoense* SCUT27, the encoding gene of *ccpA* in pMD-T*ccpA* was doubly digested with *Xba*I and *Aat*II and gel purified, then inserted into the vector pHT01 (MoBiTec, Göttingen, Germany) [23] digested by the same endonucleases. The yielded plasmid pHT01-T*ccpA* was electro-transformed into the *ccpA*-deficient strain *B*. *subtilis* MA-1 (1800 V, 25 μF, 200 Ω). Transformed cells were allowed to recover at 37°C for 3 h and subsequently spread on LB agar plates containing 5 μg/mL of chloramphenicol and incubated overnight. The strain of *B*. *subtilis* MA-1 bearing the putative *T*. *aotearoense* SCUT27 *ccpA* was denoted as *B*. *subtilis* TA-1.

#### Agar plate assay for α-amylase activity

The functional complementation test was performed using the strains of *B*. *subtilis* 168, MA-1 and TA-1. 10 μL of overnight bacterial culture was spread on LB plates containing 1.0% (*w/v*) starch and 2.0% (*w/v*) glucose as carbon sources, which was supplemented with 0.1 mM isopropyl β-d-thiogalactopyranoside (IPTG). After 2 h, 5 h and 8 h of incubation at 37°C, the plates were flooded with iodine vapor (0.5% I_2_ and 5% KI, *w/v*) [24] to visualize the starch hydrolysis halos.

#### Real time RT-PCR analysis

Real time RT-PCR was performed to study the gene expression controlled by CcpA-mediated CCR in three *B*. *subtilis* 168 derivative strains. Primers were designed using IDT website (http://www.idtdna.com/primerquest/Home/Index) to have a T_m_ between 62°C and 66°C, and an amplicon size of 100 bp (Table 2). DNA sequencing of the PCR products amplified from *B*. *subtilis* 168 genomic DNA verified the primer specificity. Saturated overnight cultures were diluted by 100-fold into fresh LB medium using a mixture of glucose and soluble starch or xylose (10 and 10 g/L, respectively) as the carbon sources, and grown at 37°C for about 6 h to reach an OD_600_ of 0.8–1.0. 1.0 OD_600_ cells were collected by centrifugation at 4°C. Total RNA was extracted by RNAprep pure kit (for cell/bacteria, TIANGEN, Beijing, China) according to the manufacturer’s instructions. cDNA was synthesized by the PrimeScript RT reagent Kit with gDNA Eraser (TaKaRa, Dalian, China) using 1 μg RNA as the template. Following synthesis, cDNA was diluted to 100 ng/μL with sterile Milli-Q water. PCR reactions contained 400 nM of each specific primers, SYBR Premix Ex Taq II (Tli RNaseH Plus), and 1 μL diluted cDNA in a final volume of 20 μL. PCR reactions were run on the LightCycler 96 (Roche, Basel, Switzerland) with a 30 s 95°C incubation step, followed by 45 cycles of 95°C (5 s) and 60°C (5 s). Sterile Milli-Q water was used as background control and 16s rRNA was designed as internal reference. Three biological repeats were performed for each gene and the results were analyzed by LightCycler 96 SW 1.0 software (Roche).

### Protein preparation

The *ccpA*, *ptsH* and *hprK* genes of *T*. *aotearoense* SCUT27 were cloned into pET30a vector to yield the expression vectors of pET-CcpA, pET-HPr and pET-HPrK containing a His-tag at C-terminus, respectively.

To obtain the CcpA protein without a His-tag, the *ccpA* gene was amplified by PCR using Tc-F and Tc-R2 as primers with the vector pMD-T*ccpA* as the template. The obtained *ccpA* gene was doubly digested with *Nde*I and *Bam*HI and then inserted into the corresponding sites of pET30a, yielding the plasmid pET-CcpA-NH.

To verify the phosphorylation site of HPr from *T*. *aotearoense* SCUT27, site-directed mutagenesis was performed to replace the Ser46 with alanine. Two PCR fragments (using *hpr*-F/hpr-MR and *hpr*-R/*hpr*-MF primer pairs, respectively, Table 2) were obtained by PCR reaction using PrimeSTAR HS DNA Polymerase. These two fragments were combined at 1:1 ratio to yield the full length gene encoding HPrM via overlapping extension PCR [25] and then inserted into pET30a to obtain pET-HPrM (between the *Nde*I and *Hin*dIII sites).

The recombinant cells *E*. *coli* BL21(DE3) containing different expression vectors were grown at 37°C to reach an OD_600_ of 0.4–0.5. Then the expression of heterologous protein was induced by 0.1 mM IPTG for 6 h at 22°C. Cell cultures were collected by centrifugation at 5,000 × g for 10 min. Cell pellets were resuspended in buffer A (20 mM sodium phosphate buffer, containing 500 mM NaCl and 20 mM imidazole, pH 7.5). Then 30 pulses of sonication (3 s each with a 3 s interval) in an ice-water bath were applied, and the sonicants were centrifuged at 10,000 × g, 4°C for 30 min for purification. The supernatant samples containing target proteins of CcpA, HPr, HPrM or HPrK/P (with a C-terminal His-tag) were purified by affinity chromatography using HiTrap^™^ Chelating HP column (GE Healthcare, Piscataway, NJ, USA), and then loaded onto a HiPrep^™^ 26/10 desalting column (GE Healthcare). Elution fractions were stored in 20 mM phosphate buffer (pH 7.5) supplemented with 100 mM NaCl and 20% (w/v) glycerol. For the surface plasmon resonance (SPR) analysis, the protein storage buffer was changed to glycerol free HBSE buffer (150 mM NaCl, 3 mM EDTA, 10 mM HEPES, pH 7.4) [7].

Sodium dodecyl sulfate-polyacrylamide gel electrophoresis (SDS-PAGE) was used to determine the purity of protein. The protein concentration was determined by a BCA Protein Assay Kit (Sangon, Shanghai, China).

### Protein-protein interaction analysis

#### *In vitro* phosphorylation of HPr

Phosphorylation of HPr was carried out in 100 mM Tris-Cl buffer (pH 7.0) supplemented with 5 mM MgCl_2_ and 10 mM NaCl. 5 mM ATP and 40 mM fructose-1,6-bisphosphate (FBP) or 40 mM glucose-6-phosphate (Glc-6-P) were selectively added into the reaction mixture to verify their functionalities. 27 μg of HPr or HPrM (to a final concentration of 50 μM) and 5 μl of HPrK/P (~0.15 μg) were mixed with the reaction buffer to a final volume of 50 μl and incubated at 37°C for 10 min. The reaction was stopped by heating at 70°C for 5 min. About 0.5 μg of native and phosphorylated samples were loaded on SDS-PAGE with 100 μM MnCl_2_ and 20 μM Phos-tag^™^ solution purchased from Boppard (Guangzhou, China). The phosphorylated form of HPr was detected by its slower migration on Phos-tag^™^ SDS-PAGE, because the phosphate group binding with Phos-tag would slow down the corresponding protein’s mobility [26, 27].

### Determination of protein binding via affinity chromatography

*E*. *coli* cells containing the expressed *T*. *aotearoense* CcpA without His-tag were resuspended in buffer A and lysed by sonication using the same method as mentioned above. After cell disruption, the lysates were centrifuged and the supernatants were heated at 65°C for 30 min followed by a centrifugation at 5000 rpm for 20 min. 2 mg of phosphorylated HPrSerP containing a C-terminal His-tag was mixed with the partially purified CcpA and incubated with 20 mM FBP at 37°C for 30 min. If necessary, 0.6% formaldehyde was added into the binding mixture. The samples were loaded onto a HiTrap^™^ Chelating HP column. The fractions containing HPrSerP were collected. The wash-out fractions were removed the crosslinks in boiling water bath for 5 min and detected by 12% SDS-PAGE. The protein after affinity was identified by LC/MS on a LTQ Orbitrap XL mass spectrometer (Thermo Fisher Scientific, Bremen, Germany). The methods of sample treatment and MS were as described by Sun *et al*. [28]. Subsequent MS analysis was performed in Matrix Science website (http://www.matrixscience.com/), and NCBInr was chosen as searching database. Peptide mass tolerance (MS) was set to ± 50 ppm (# ^13^C = 1), and fragment mass tolerance was set to ± 0.5 Da.

### Determination of protein binding via surface plasmon resonance

SPR (surface plasmon resonance) analysis of the purified CcpA, HPr, HPrSerP and HPrM was performed using SPRi system (Plexera, V3 version, Seattle, WA) at 25°C as described by Foudeh *et al*. [29]. All processes were operated according to the Plexera’s instructions. In brief, for the analysis of protein-protein interactions, CcpA was printed as spots on the Gold-coated sensor slides by amine coupling with three technical repeats. PBST buffer (137 mM NaCl, 2.7 mM KCl, 10 mM Na_2_HPO_4_, 2 mM KH_2_PO_4_ with 0.05% Tween 20, pH 7.2) was used as a running buffer during immobilization and interaction analyses. Different titrations of HPr, HPrSerP or HPrM were treated as analysts to flow through the surface of slide at a flow rate of 2 μL/s. The association and dissociation duration were both 300 s. The biomolecular binding was monitored in real-time with the Plexera SPRi system. BSA (Bovine Serum Albumin) purchased from TaKaRa was used as a reference.

## Results and Discussion

### CCR analysis of *T*. *aotearoense* SCUT27

2-DG is a glucose analogue which is best known as an inhibitor of glucose metabolism [30]. In glycolysis, it is converted to phosphorylated 2-DG by hexokinase [31]. However, the phosphorylated 2-DG cannot be catalyzed by phosphoglucose isomerase in further glycolysis process [32, 33], because the 2-hydroxyl group was replaced by hydrogen in 2-DG. 2-DG can be uptaken by the glucose transporters [31, 34]. Therefore, cells with higher glucose uptake also have a higher uptake of 2-DG. Since 2-DG hampers microorganism’s growth, it has been widely used as an inducer of carbon catabolite repression [5, 35–37]. We added 0.5 g/L 2-DG to different culture media to detect the CCR by monitoring the culture optical densities.

**Fig 1:**
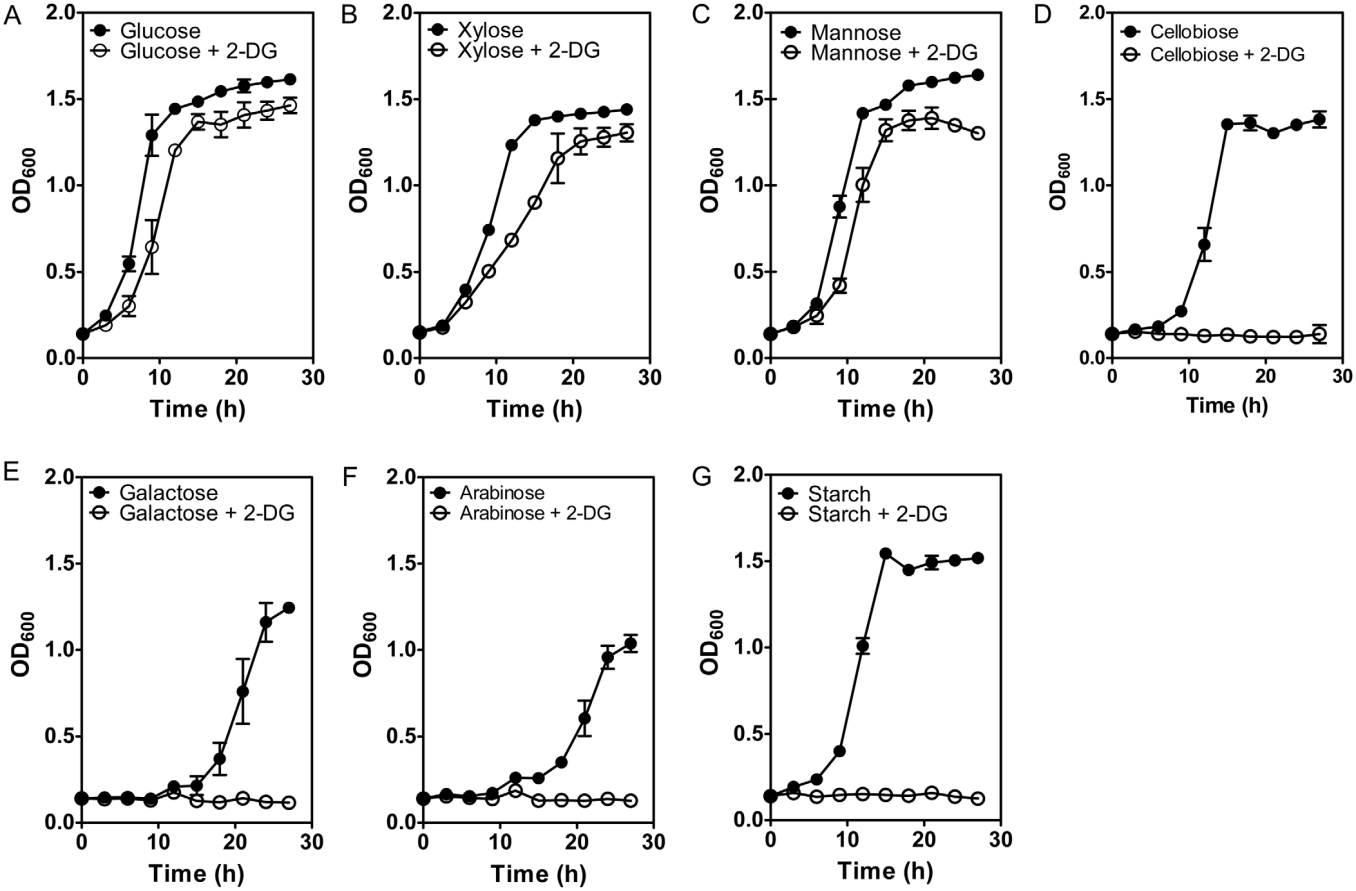
Effects of 2-DG on the cell growth for *T*. *aotearoense* SCUT27 using different sugars as the main carbon source. 0.5 g/L of 2-DG was added to the culture medium containing 5 g/L of glucose (A), xylose (B), mannose (C), cellobiose (D), galactose (E), arabinose (F) or starch (G). Data were plotted as mean ± SD (n = 2).

When supplied with 5 g/L glucose, xylose or mannose as the main carbon source, the addition of 0.5 g/L 2-DG extended the lag phases, but only reduced the final cell densities of *T*. *aotearoense* SCUT27 by 10–15% (Fig 1A–1C). On the contrary, for fermentations using cellobiose, galactose, arabinose and starch as the main carbon source, no obvious cell growth was observed when 0.5 g/L 2-DG was added into the culture medium (Fig 1D–1G). These results indicated that 0.5 g/L 2-DG could lead to a strong CCR of cellobiose, galactose, arabinose and starch. However, SCUT27 efficiently metabolized xylose and mannose with the presence of 0.5 g/L 2-DG, suggesting an absence of CCR. A study of the glycobiome of the TGPAs-related *Thermoanaerobacter* sp. X514 revealed that glucose, xylose, fructose, and cellobiose metabolism were each featured in distinct functional modules. The transport systems of glucose and xylose were regulated by transcriptional antiterminators of the BglG family and the co-utilization of glucose and xylose was interpreted as a lack of CCR [15]. While for another TGPA strain, researchers demonstrated that obvious cAMP-independent CCR of cellobiose utilization existed in *T*. *saccharolyticum* [5].

**Fig 2:**
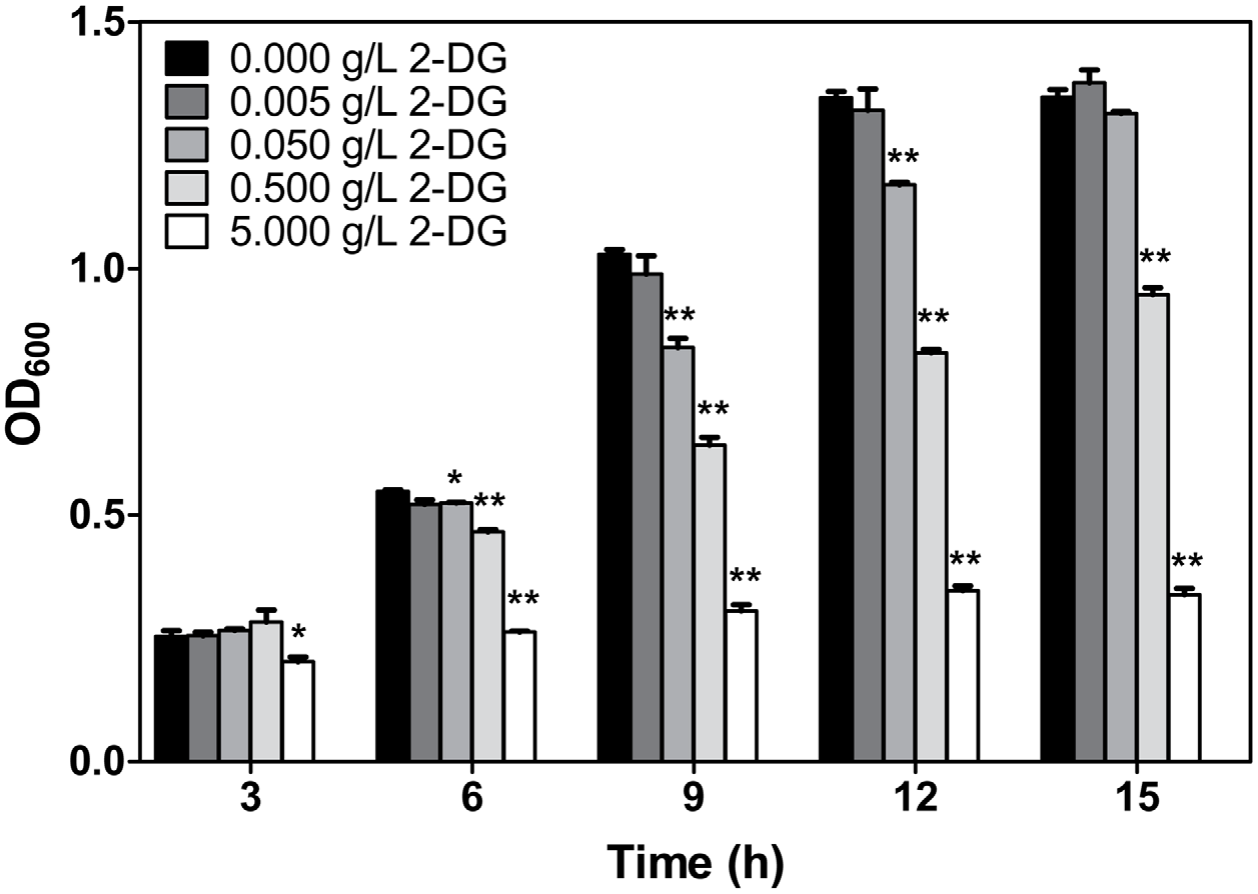
The inhibitory effects of 2-DG concentration on xylose-related CCR of *T*. *aotearoense* SCUT27. Overnight cultures were inoculated into fresh medium containing 5 g/L xylose supplemented with increasing concentrations of 2-DG (0, 0.005, 0.05, 0.5 or 5 g/L). After incubation at 55°C for different hours, culture optical densities were measured and expressed as mean ± SD (n = 2). *, significant difference at *p*<0.05 (T-test); **, very significant difference at *p*<0.01.

It is noted that the delay of growth caused by the addition of 0.5 g/L 2-DG was more significant in fermentation using xylose than that using glucose or mannose as carbon source (Fig 1A–1C). The effects of 2-DG on SCUT27 growth were studied in cell cultures grown on 5 g/L of xylose in the presence of different concentrations of 2-DG (Fig 2). When 0.005 g/L 2-DG was added, the cell growth pattern was almost same as the control (without the 2-DG addition). With the increase of 2-DG concentration, the inhibition on SCUT27 cell growth was strengthened remarkably. When the 2-DG concentration reached the same level as xylose (5 g/L), the utilization of xylose was completely repressed. However, if the xylose concentration was 100-fold more than 2-DG, the repression of xylose utilization turned to be less obvious, indicating that the growth inhibition of 2-DG was competitively reversible by adding particular substrate [33].

**Fig 3:**
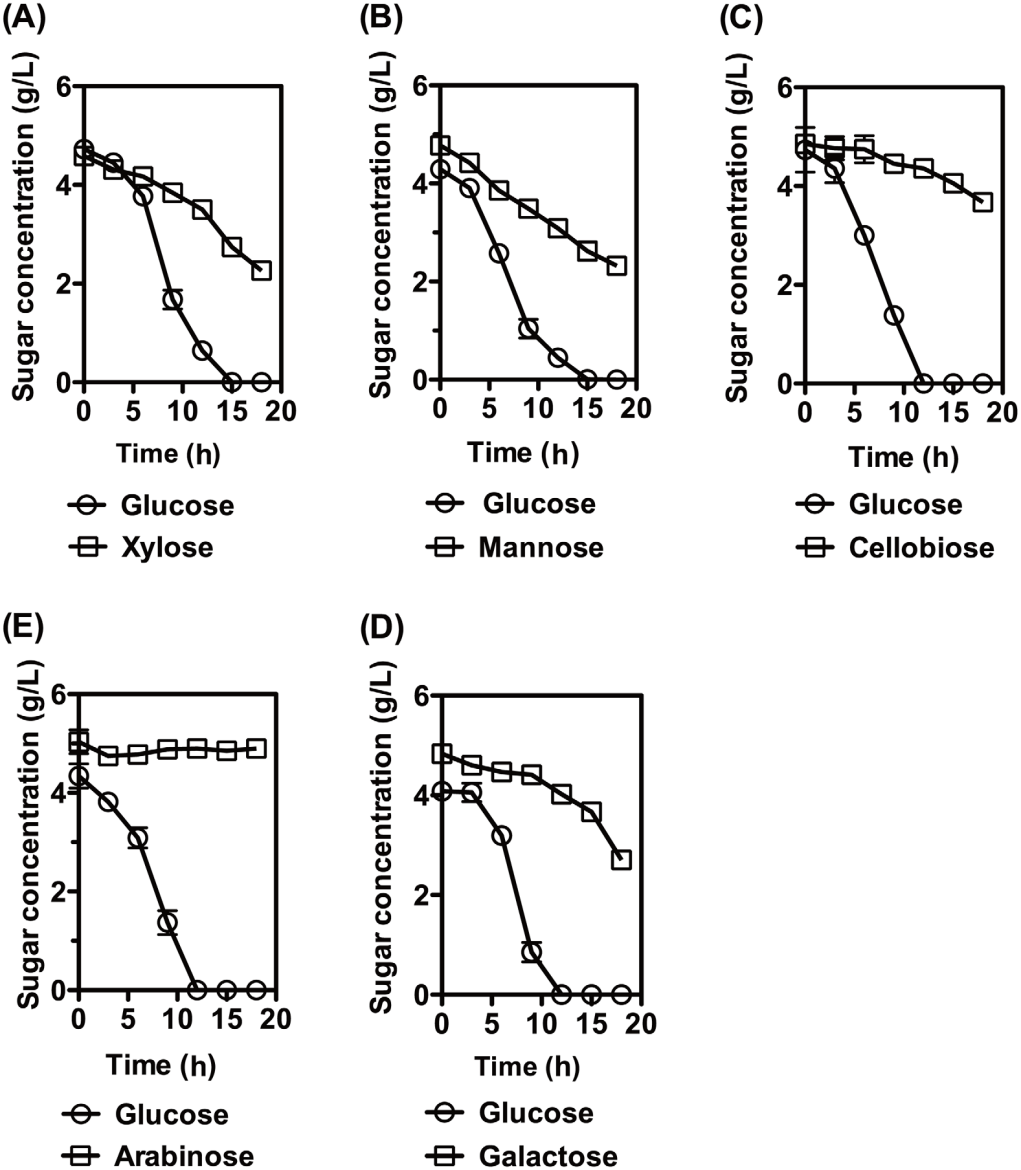
Glucose inhibition assays on carbohydrates. *T*. *aotearoense* SCUT27 was cultured at 55°C in serum bottle containing 50 mL medium using mixed sugar as carbon sources (5 g/L glucose and 5 g/L different sugar). (A), xylose (B), mannose (C), cellobiose (D), galactose and (E), arabinose. Carbohydrate measurements were performed in duplicates.

To better understand the effects of glucose on other carbohydrate utilization, sugar consumptions were investigated when *T*. *aotearoense* SCUT27 was cultured in the medium containing mixed carbon sources (Fig 3). Time profiles demonstrated that the glucose existence could apparently inhibit the metabolism of galactose, arabinose and cellobiose. In contrast, xylose or mannose could be simultaneously utilized with glucose by *T*. *aotearoense* SCUT27. The results were completely consistent with our previous studies on sugar utilization by *T*. *aotearoense* SCUT27 [16–18]. It’s universally recognized that the pleiotropic transcription factor CcpA, HPr protein in the PTS system and HPrK/P play the key roles in the firmicute bacteria [4, 8, 38]. Thus, we deduced that the CCR in *T*. *aotearoense* SCUT27 was HPr, HPrK/P and CcpA related as many other firmicutes.

### Cloning of *ccpA*, *ptsH* and *hprK* genes from *T*. *aotearoense* SCUT27

**Fig 4:**
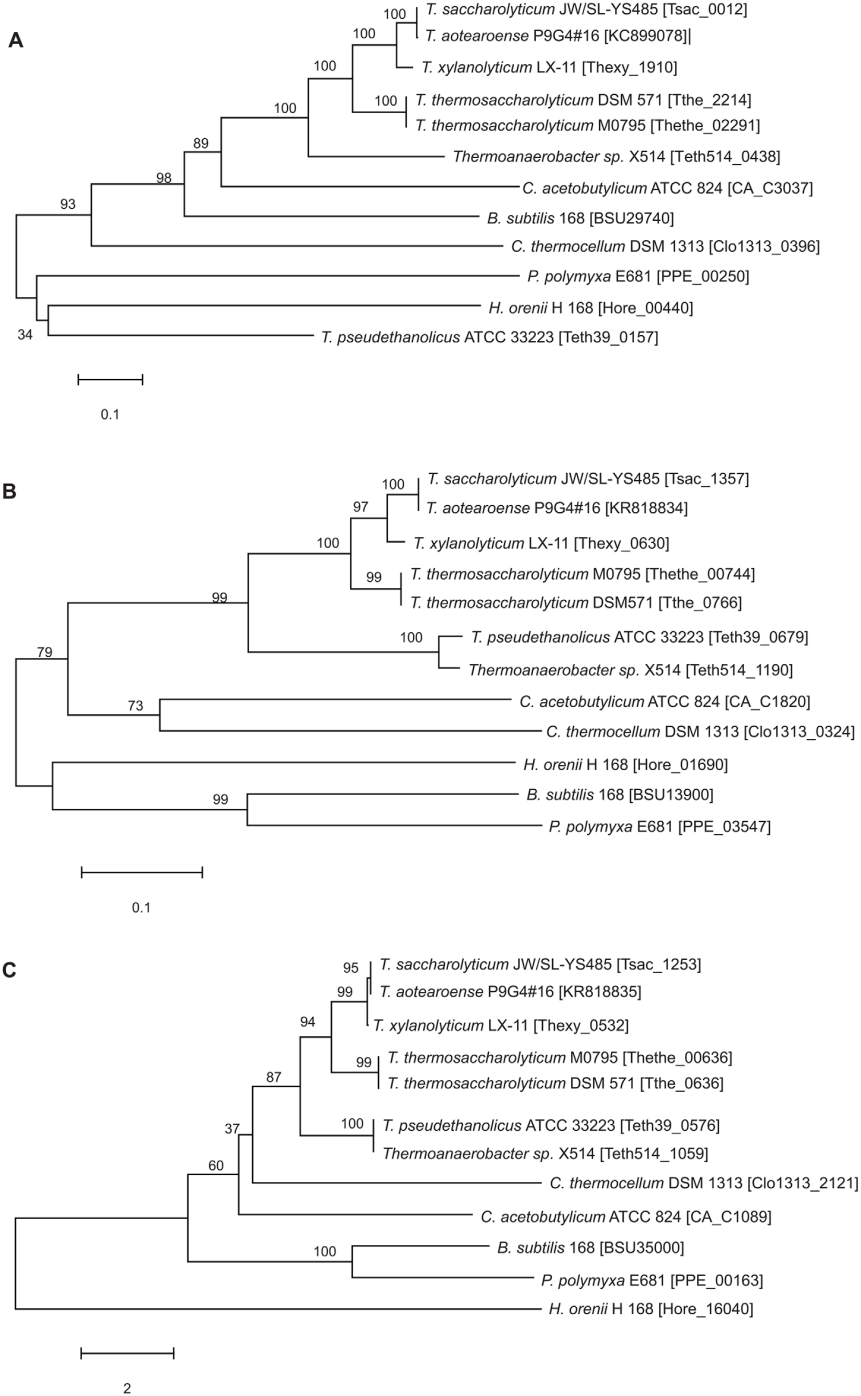
The phylogenetic trees of *ccpA* (A), *ptsH* (B) and *hprK* (C) from *T*. *aotearoense* SCUT27. The phylogenetic trees were constructed by Neighbor-Joining (NJ) method with 1000 replicates of bootstrap test using MEGA4.0. The numbers for gene “locus tag” on the chromosome of some strains in the GenBank were shown.

Alignment of the putative *ccpA*, *ptsH* and *hprK* genes from five TGPAs (*Thermoanaerobacter* sp. X514, CP000923; *Thermoanaerobacterium thermosaccharolyticum* DSM 571, CP002171; *Thermoanaerobacterium saccharolyticum* JW/SL-YS485, CP003184; *Thermoanaerobacterium xylanolyticum* LX-11, CP002739 and *Thermoanaerobacterium thermosaccharolyticum* M0795, CP003066) showed that there are about 25 bp conserved regions presented at their 5' and 3' of the targeted genes, respectively, except the *Thermoanaerobacter* sp. X514 (S1 Fig). Based on the conserved sequences, primers of *ccpA*-F/*ccpA*-R, *hpr*-F/*hpr*-R and *hprK*-F/ *hprK*-R (Table 2) were designed and used to amplify the corresponding genes. The generated PCR products were cloned into pMD^®^18-T and sequenced separately. The nucleotide sequences of these genes have been deposited in the GenBank database under the accession number KC899078 (1011 bp), KR818834 (267 bp) and KR818835 (915 bp) for *ccpA*, *ptsH* and *hprK*, respectively. The sequenced products could be translated into ORFs of 336, 88 and 304 amino acids beginning with start codon and ended by TAA. The protein products have the theoretical PIs and molecular weights of 5.69, 37.2 kDa, 7.07, 11.1 kDa, and 5.92, 36.0 kDa, corresponding with *ccpA*, *ptsH* and *hprK*. The phylogenetic trees of these three genes were showed in Fig 4. The closest phylogenetic relative was *T*. *saccharolyticum* JW/SL-YS485 with identities of about 99%, followed by the strains of *T*. *xylanolyticum* LX-11, *T*. *thermosaccharolyticum* DSM571 and *T*. *thermosaccharolyticum* M0795. Protein-protein BLAST search showed that the encoded proteins share higher amino acid sequence similarities with almost ten proteins annotated as CcpA, HPr and HPrK/P in the whole-genome sequencing data. However, the characterization of the similar proteins has not been experimentally verified so far.

### Functional complementation of CcpA in *B*. *subtilis*

**Fig 5:**
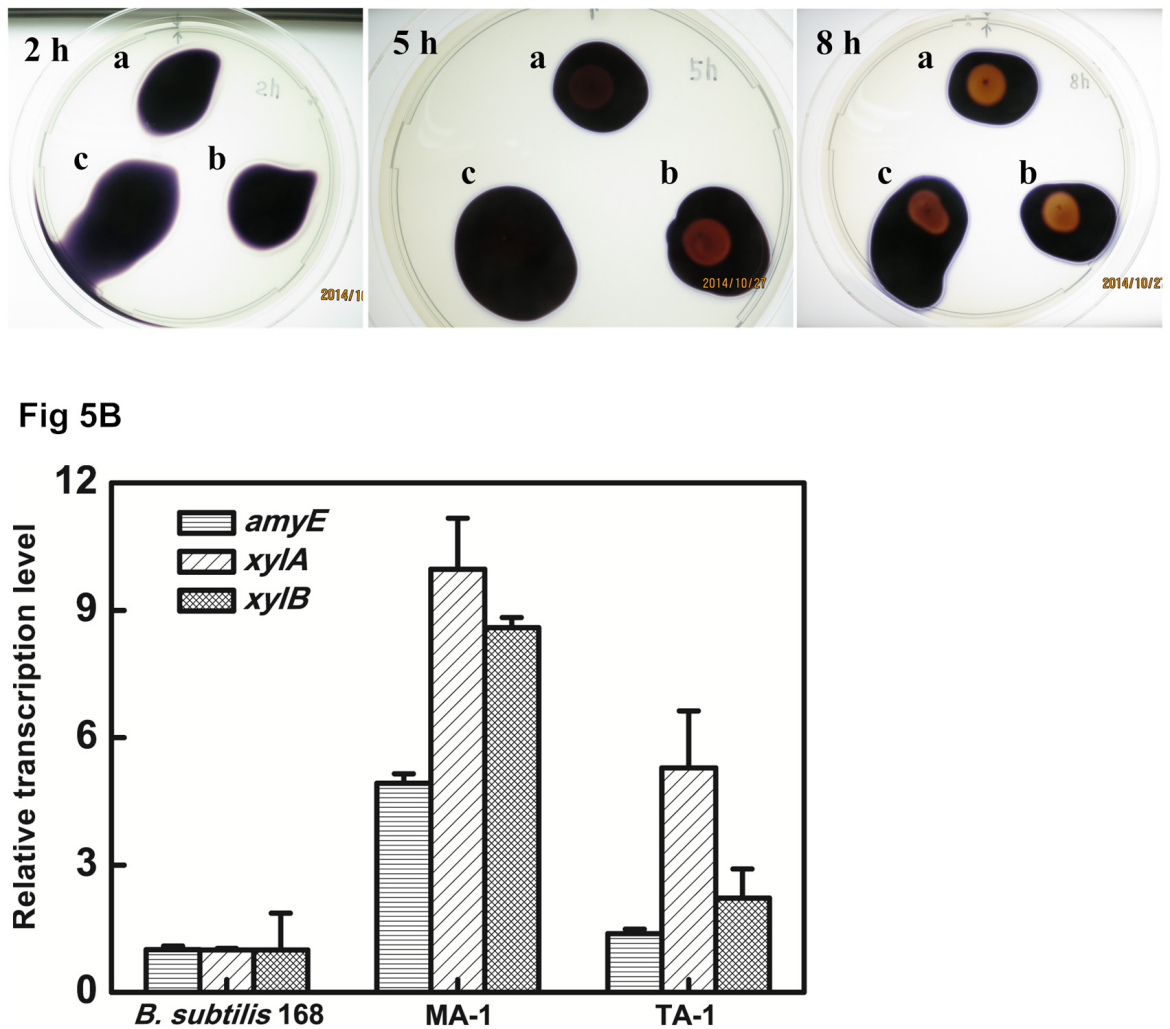
Functional verification of *ccpA* from *T*. *aotearoense* SCUT27 in *B*. *subtilis* mutant MA-1, which was defective in the *ccpA* gene (BSU29740). (A) Starch utilization of *B*. *subtilis* 168 (a), MA-1 (b) and TA-1 (c) on agar plates containing 1% (*w/v*) starch and 2% (*w/v*) glucose. After the plates incubated at 37°C for 2 h, 5 h and 8 h, the plates were flooded with I_2_/KI (0.5:5.0%, *w/v*) solution to examine the starch hydrolysis halos. (B) Comparison of expression levels of the *amyE*, *xylA* and *xylB* genes. The bars represent the mean values of the expression levels of different genes. All the experiments were done in triplicate and statistically tested (T-test).

It was reported that CcpA is involved in the catabolite repression of amylase gene expression in *Bacillus subtilis* (*B*. *subtilis*) [39]. To verify the function of the cloned *ccpA* from *T*. *aotearoense* SCUT27, functional complementation was carried out in the *ccpA*-deficient mutant, *B*. *subtilis* MA-1 (Fig 5A) [6]. After incubation at 37°C for 5 h, MA-1 showed a clearly lighter hydrolysis halo than *B*. *subtilis* 168 on the starch containing plate. No hydrolysis halo was observed for the wild-type strain, indicating the existence of glucose inhibited the starch utilization. When the *ccpA* in the chromosome of *B*. *subtilis* 168 was deleted to yield the MA-1, an obvious hydrolysis halo appeared, indicating that the amylase expression inhibition by glucose was released. And there were not any signs of starch utilization at this time by the TA-1 strain, indicating that the introduced *ccpA* from SCUT27 functioned as the pleiotropic regulator CcpA in *B*. *subtilis* MA-1. After 8 h, apparent hydrolysis halos were found in all three strains, suggesting that the glucose in bacterial spread area had been exhausted, and starch started to be metabolized. Next, we compared the relative expression levels of *amyE* (BSU03040), *xylA* (BSU17600) and *xylB* (BSU17610) genes, encoding α-amylase, xylose isomerase and xylulose kinase, which have been clearly demonstrated to be repressed by CcpA-mediated CCR in *B*. *subtilis* [40–42]. As shown in Fig 5B, the expression levels of *amyE*, *xylA* and *xylB* in MA-1 were expectedly higher (*p*<0.01) compared with the wild type strain when cells were grown on starch or xylose with glucose, suggesting that the CCR repressed for these genes. When the pHT01-T*ccpA* was electro-transformed into the strain MA-1, the transcription level of each of these genes in the resulting strain TA-1 was significantly lower than that in MA-1 (*p*<0.01). These results indicated that the genes of *amyE*, *xylA* and *xylB* restored CCR in the *B*. *subtilis ccpA*-disruptive strain when the SCUT27 *ccpA* was expressed in the strain MA-1. Through sequence searching using WTGNNARCGNWWWCAW as template [7], catabolite responsive elements (*cre*) were specified as TGTAAGCGTTAACAA and TGGAAGCGCAAACAA at -118, +48 bp for *amyE* and *xylA* from the chromosome of *B*. *subtilis* 168 (GenBank: AL009126), respectively. Here, the first base of each gene was set as +1. No typical *cre* sequence was observed within 1000 bp upstream or downstream of *xylB*. Because *xylA* and *xylB* are neighbourhood genes on the chromosome, they might share the same *cre* sequence. Thus, the expression of *xylB* might be regulated by CcpA in *B*. *subtilis* 168. All these data supported that the cloned *ccpA* from SCUT27 encodes an active CcpA.

### Functional characterization of HPr and HPrK/P

**Fig 6:**
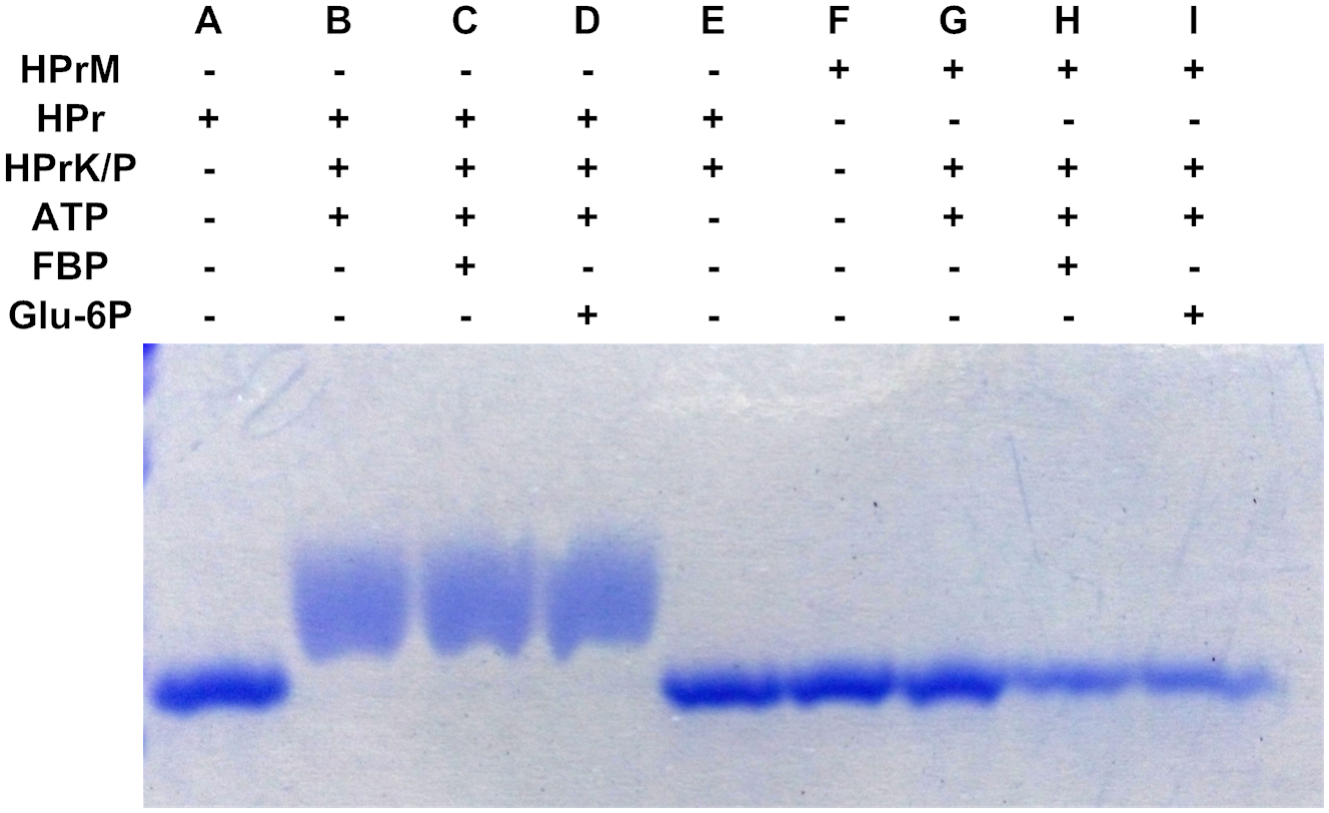
Phos-tag^™^ PAGE assay of HPr phosphorylation by HPrK/P. 27 μg of HPr or HPrM and 5 μl of HPrK/P were mixed in 50 μl 100 mM Tris-Cl buffer (pH 7.0) containing 5 mM MgCl_2_, 10 mM NaCl and incubated at 37°C for 10 min. 5 mM ATP, 40 mM FBP and 40 mM Glc-6-P were selectively added into the reaction mixture. 0.5 μg protein samples were load on Phos-tag^™^ SDS-PAGE.

Binding of HPr to CcpA requires its phosphorylation by HPrK/P at the Ser-46 [4, 43, 44]. To detect the activity of HPrK/P, the recombinant HPr and HPrK/P were incubated with 5 mM ATP for 10 min. The product analysis showed that HPr was phosphorylated by HPrK/P with the existence of ATP judged by its migration on Phos-tag^™^ SDS-PAGE (Fig 6A and 6B) [26]. HPrK/P kinase activity was reported to be stimulated by FBP or Glc-6-P in Gram-positive bacteria [4, 45, 46]. However, FBP or Glc-6-P was not essential for HPrK/P kinase activity from *T*. *aotearoense* SCUT27 in this study (Fig 6C and 6D). Some researchers made the same observation that FBP or Glc-6-P could be omitted for highly purified recombinant HPrK/P in *Enterococcus faecalis* [26] and *Streptococcus* sp. [47]. HPrK/P lost its kinase activity when there was no ATP in the reaction mixture, indicating that it was ATP-dependent (Fig 6E). In order to investigate the specificity of HPrK/P, we introduced the serine 46 to alanine mutation in the HPr. No phosphoryltion of HPrM was observed on the non-denaturing PAGE (Fig 6F–6I), indicating the ATP-dependent protein kinase of HPrK/P specifically phosphorylated the 46 seryl residue in HPr.

### Interaction analysis of CcpA and HPrSerP

**Fig 7:**
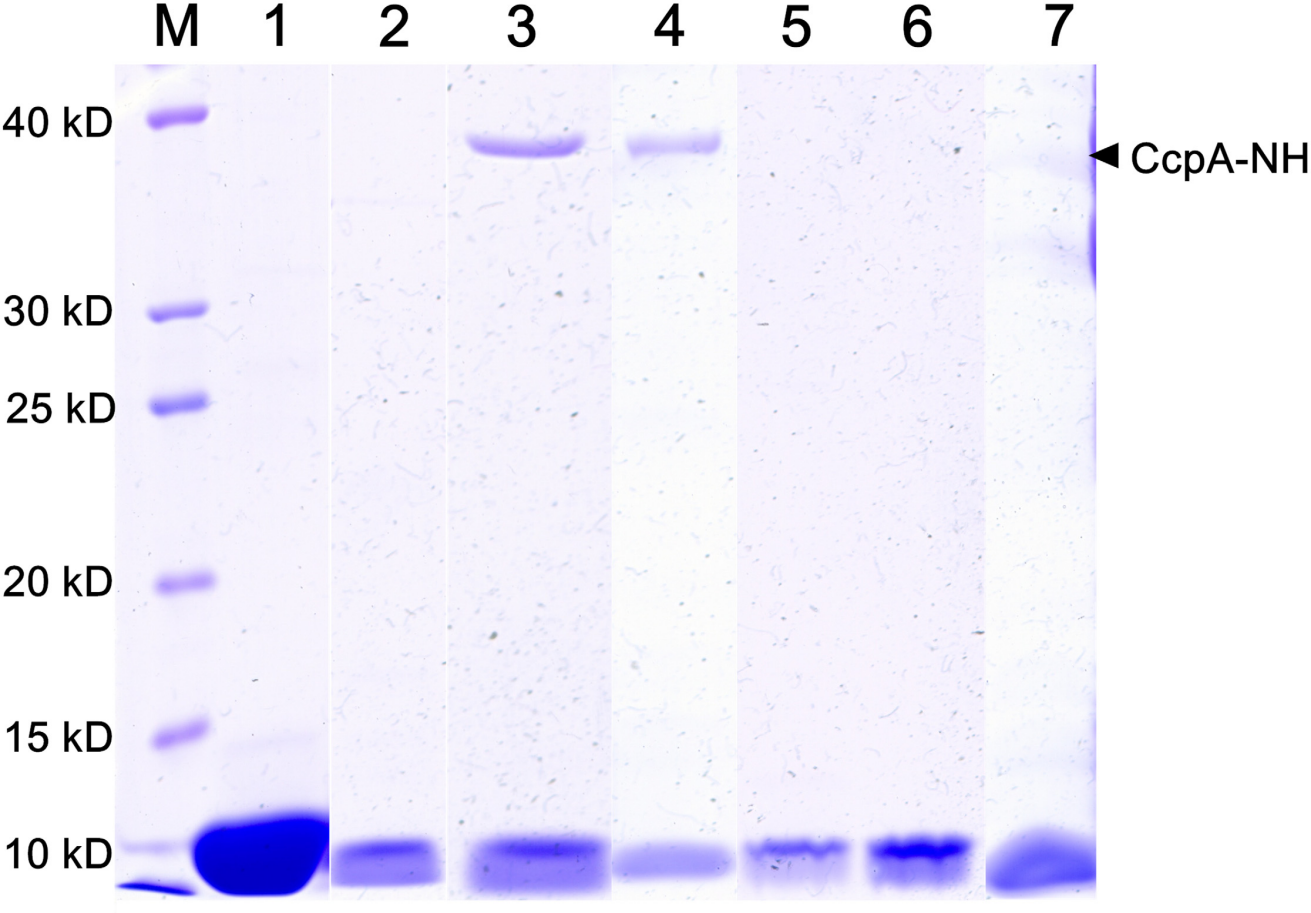
Interaction analysis of CcpA with HPr. The affinity purified HPr and HPrM were phosphorylated with HPrK/P as previously described. Heat-treated cell extracts containing CcpA-NH mixed with different protein samples were loaded onto the HiTrap^™^ Chelating HP column. The eluted fractions were separated by SDS-PAGE using a 12% acrylamide gel and stained with coomassie brilliant blue. M: protein standards, 1: purified HPr, 2: HPrSerP, 3: CcpA-NH mixed with HPrSerP, 4: CcpA-NH mixed with HPrSerP and formaldehyde, 5: CcpA-NH mixed with HPr, 6: CcpA-NH mixed with HPrM, 7: Nitrilase mixed with HPrSerP as negative control.

CcpA and HPrSerP interactions play essential roles in regulating the CCR in firmicutes [4, 7]. In an attempt to characterize the binding of CcpA with HPrSerP, the HPr or HPrSerP containing the C-terminal hexahistine-tag was mixed with cell extracts containing heat-treated CcpA-NH and incubated at 37°C for 30 min. The mixtures were subjected to the HiTrap^™^ Chelating HP column. The eluted protein samples were boiled for 5 min to reverse the crosslinks, and then analyzed by SDS-PAGE (Fig 7). For the non-phosphorylated forms of HPr and HPrM, only one protein band in 11.1 kD was detected as expected. On the contrary, a band that migrates at a molecular weight similar to that of CcpA was detected in elutes along with HPrK/P treated HPr. This band was further confirmed to be a CcpA-homologous protein of *T*. *aotearoense* SCUT27 by LC/MS (S1 File). All these results suggested that the CcpA was only crosslinked with Ser46 phosphorylated HPr. To be noted, interaction of CcpA with HPrSerP was also observed in the absence of formaldehyde, which was different to studies reported by Wünscheu *et al*. [25]

The interaction of HPr and CcpA was also detected by SPR analyses to determine the equilibrium binding constants (*K*_D_), in which the purified CcpA was covalently coupled to a Gold-coated sensor slide. BSA was used as control and showed no binding with any proteins. 5 to 25 μM of HPrSerP was used to bind CcpA and the results showed specific binding with rapid association and slow dissociation phases. An equilibrium analysis by Langmuir fits of plots yielded the *K*_D_ of 2.22 ± 0.36 nM. The HPr without HPrK/P treatment and HPrM were also injected. The association (*k*_a_) and dissociation rate constants (*k*_d_) of the HPr-CcpA and HPrM-CcpA were calculated by fitting the sensorgrams according to Langmuir binding model (Table 3). Results showed minor differences between association rate constants of these protein complexes. However, the dissociation rate constants for HPr-CcpA and HPrM-CcpA were much higher than that for HPrSerP-CcpA, indicating significantly faster dissociation happened. All these results were consistent with the previous studies.

**Table 3:** Association and dissociation rate constants and equilibrium binding constants ^a^.

Protein	*k*_a_ (M^-1^×s^-1^)	*k*_d_ (s^-1^)	*K*_D_ (M)
HPrSerP	3.50 ± 0.28×10^2^	7.78 ± 0.59×10^−7^	2.22 ± 0.36×10^−9^
HPr	4.03 ± 0.12×10^2^	9.22 ± 1.15×10^−4^	2.29 ± 0.57×10^−6^
HPrM	9.58 ± 2.23×10^1^	5.76 ± 1.89×10^−4^	6.01 ± 0.94×10^−6^

^a^ The purified CcpA was covalently coupled to Gold-coated sensor slides, and different protein samples were flowed through the chip.

In conclusion, xylose and mannose were competitively repressed by glucose analogue (2-DG), which were in different functional ways from cellobiose, galactose, arabinose and starch in *T*. *aotearoens*e SCUT27. Gene cloning and protein functional characterization confirmed that T. aotearoense SCUT27 has *ccpA*, *ptsH* and *hprK* genes. Interaction analysis showed that CcpA and HPrSerP were specifically bound with high affinity. Understanding the components of CCR is critical to the development of fermentation with wider feedstock utilization. Further study and genetic manipulation of *T*. *aotearoense* SCUT27 should help the strain in expanding carbon source utilization profiles to produce bio-based chemicals.

## Supporting Information

S1 Fig Gene sequence alignments of *ccpA* (A), *hpr* (B) and *hprk* (C) from TGPAs. (*Thermoanaerobacter* sp. X514, CP000923; *Thermoanaerobacterium thermosaccharolyticum* DSM 571, CP002171; *Thermoanaerobacterium saccharolyticum* JW/SL-YS485, CP003184; *Thermoanaerobacterium xylanolyticum* LX-11, CP002739 and *Thermoanaerobacterium thermosaccharolyticum* M0795, CP003066).
(PDF)

S1 File LC/MS analysis report: MASCOT Search Results. MS analysis was performed in Matrix Science website, and all information related to target protein is highlighted.
(PDF)
