# Supplementary material for: Carbon Catabolite Repression and the Related Genes of ccpA, ptsH and hprK in Thermoanaerobacterium aotearoense
Source: PLoS One. 2015 Nov 5;10(11):e0142121. doi: 10.1371/journal.pone.0142121 (PMC4634974; doi:10.1371/journal.pone.0142121)

S1 Fig. Gene sequence alignments of *ccpA* (A), *hpr* (B) and *hprk* (C) from TGPAs

A

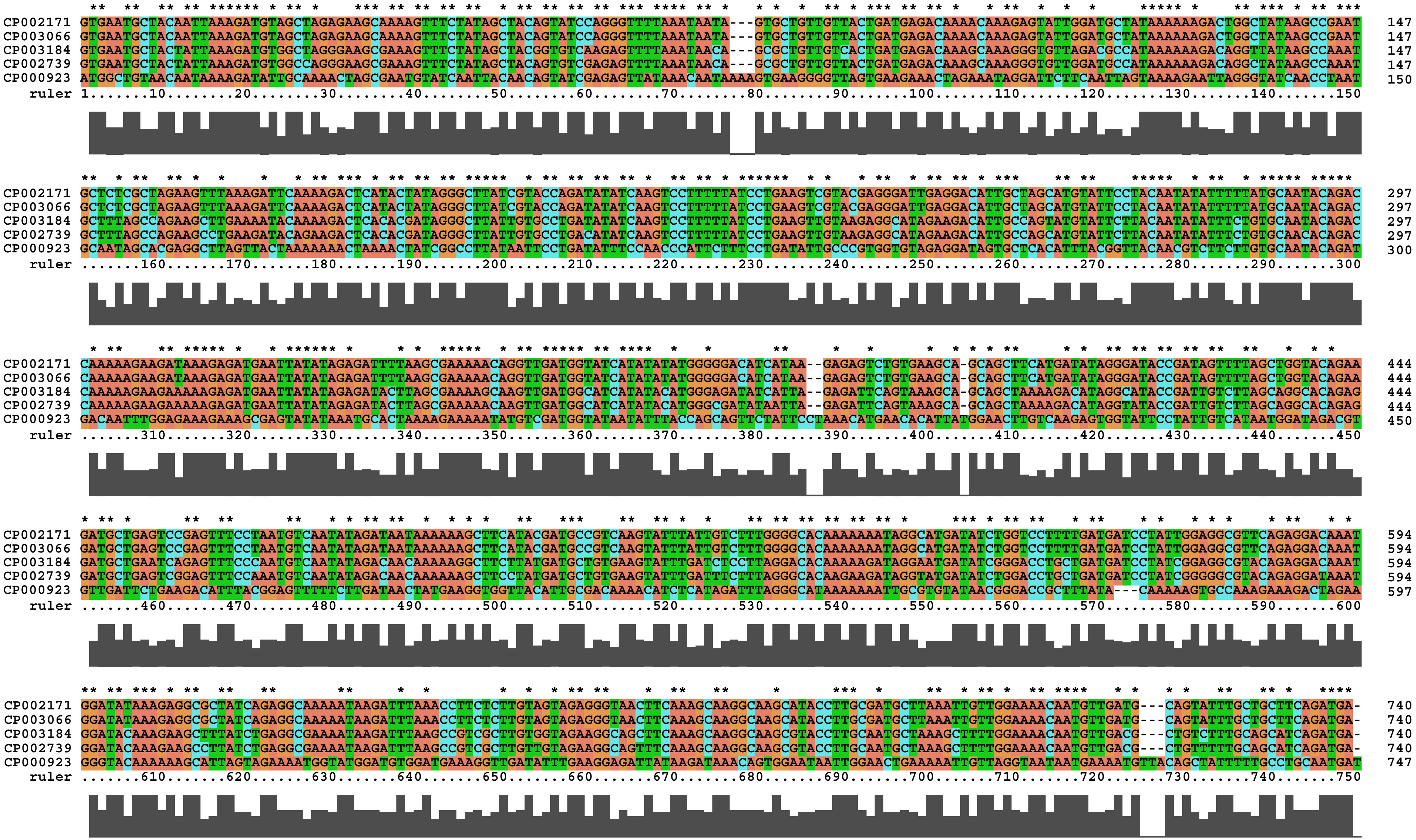

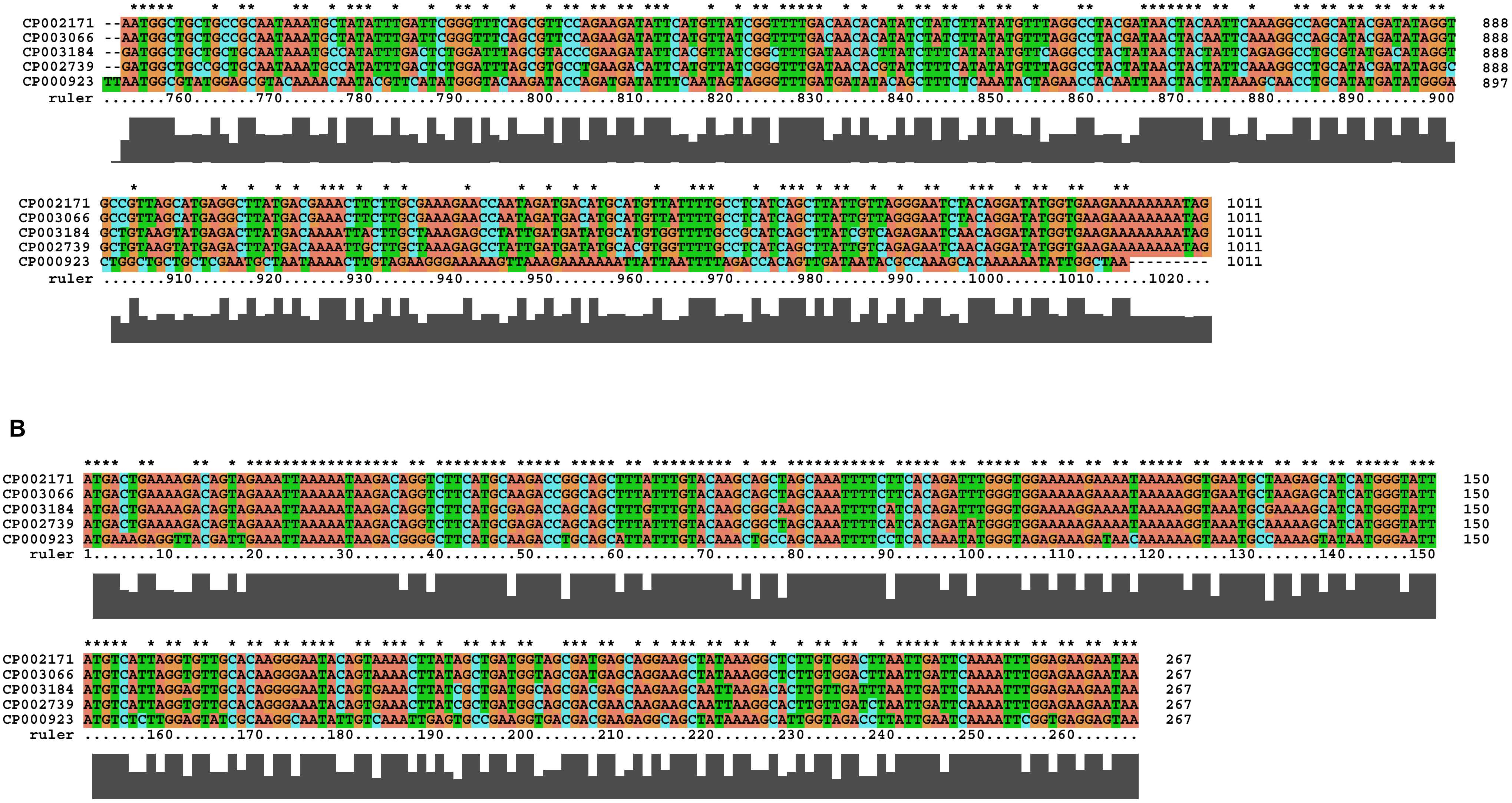
B
CP002171
CP003066
CP003184
CP002739
CP000923
ruler

C

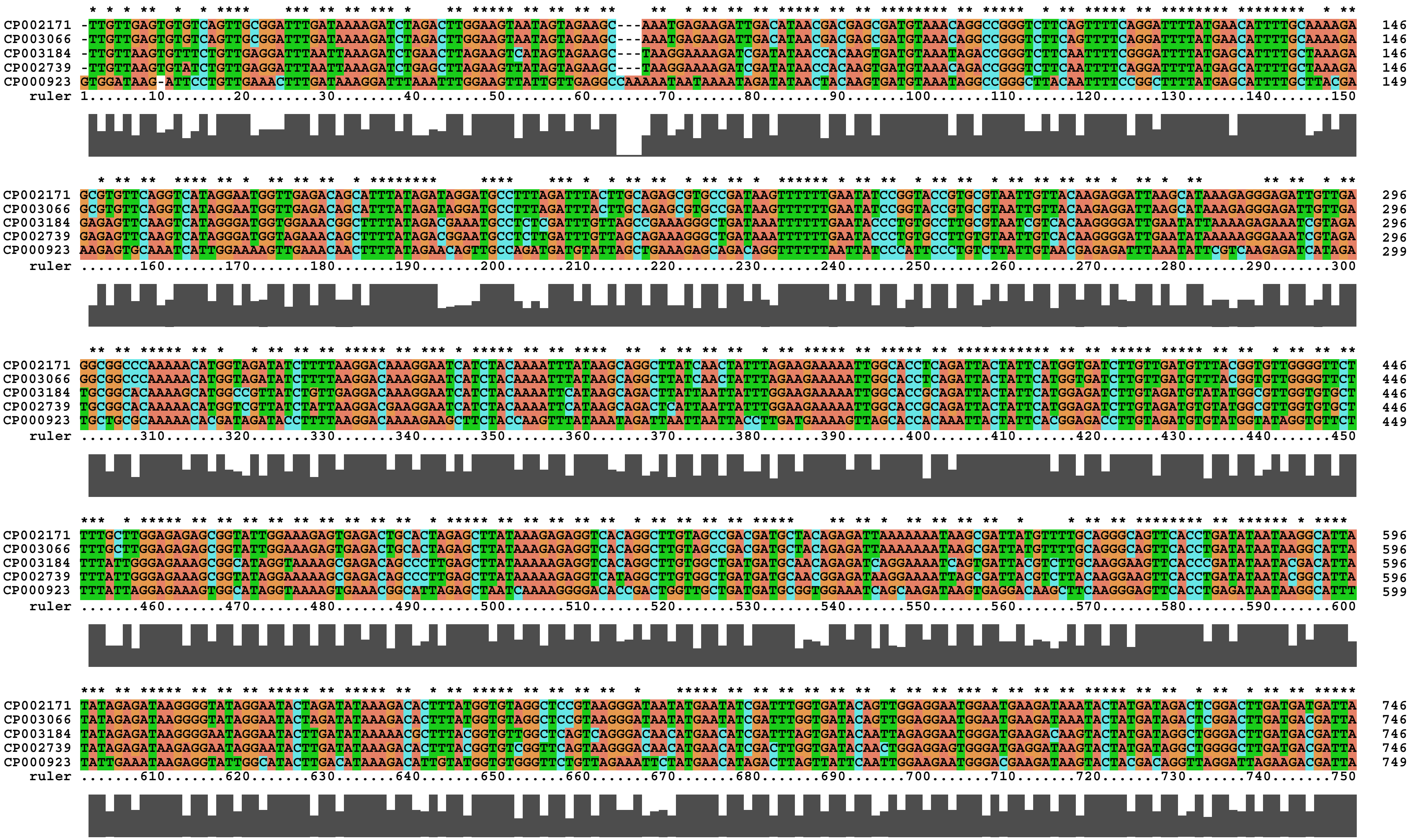
CP002171
CP003066
CP003184
CP002739
CP000923
ruler
146
146
146
146
149
296
296
296
296
299
446
446
446
446
449
596
596
596
596
599
746
746
746
746
749

```
           ** ** ***** **  * ***********  * ** *********** ** **  * ***** ** ******** ** ** ** ** ***** ** ** ** ** ******** ** ** ***** ** ** ***** ******** **
CP002171 CATAAAGTTTTTGGACATCAAAGTTCCAAAACTGACAATACCTGTAAGACCAGGAAGGAATCTTGCGATAATAGTGGAGGTTGCAGCGATGAACCATAGACAAAAGCAAATGGGATATAATGCTGCACACGAGCTTAACAAAAAATTGTT   896
CP003066 CATAAAGTTTTTGGACATCAAAGTTCCAAAACTGACAATACCTGTAAGACCAGGAAGGAATCTTGCGATAATAGTGGAGGTTGCAGCGATGAACCATAGACAAAAGCAAATGGGATATAATGCTGCACACGAGCTTAACAAAAAATTGTT   896
CP003184 CATAAAGTTTTTAGACATAAAAGTTCCAAAATTGACTATACCTGTAAGGCCAGGGCGAAATCTTGCCATAATAGTTGAAGTCGCGGCCATGAATCATAGGCAGAAACAAATGGGTTACAACGCTGCCCATGAGCTTAATAAAAAATTGTT   896
CP002739 CATAAAGTTTTTAGACATAAAAGTTCCAAAGCTGACTATACCTGTAAGACCGGGGCGAAATCTTGCCATAATAGTGGAAGTGGCAGCCATGAATCACAGGCAAAAGCAAATGGGCTATAACGCTGCTCATGAGCTTAATAAAAAATTGTT   896
CP000923 TATCAAATTTTTAGATGTCAAAGTTCCAAAGCTTACTATACCTGTAAGACCAGGAAGAAATCTCGCGATAATAGTAGAAGTAGCAGCGATGAACCACAGGCAAAAACAAATGGGATACAACGCTGCTCACGAACTTAACAAAAAATTATT   899
   ruler .......760.......770.......780.......790.......800.......810.......820.......830.......840.......850.......860.......870.......880.......890.......900
```

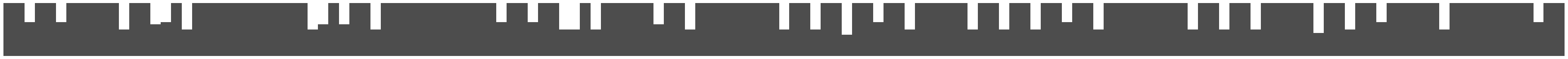

```
          ***** **    *  ***
CP002171 GAAACAAATTTCAACTTAA   915
CP003066 GAAACAAATTTCAACTTAA   915
CP003184 GAAACAGATTTCCACATAA   915
CP002739 GAAACAGATTTCCACATAA   915
CP000923 AAAACAAATAGGGAATTAA   918
   ruler .......910.........
```

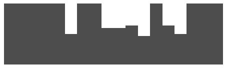

Supplement: S1 Fig — (Thermoanaerobacter sp. X514, CP000923; Thermoanaerobacterium thermosaccharolyticum DSM 571, CP002171; Thermoanaerobacterium saccharolyticum JW/SL-YS485, CP003184; Thermoanaerobacterium xylanolyticum LX-11, CP002739 and Thermoanaerobacterium thermosaccharolyticum M0795, CP003066). (PDF) [file pone.0142121.s001.pdf]
