# Supplementary material for: Carbon Catabolite Repression and the Related Genes of ccpA, ptsH and hprK in Thermoanaerobacterium aotearoense
Source: PLoS One. 2015 Nov 5;10(11):e0142121. doi: 10.1371/journal.pone.0142121 (PMC4634974; doi:10.1371/journal.pone.0142121)
Supplement: S1 File — MS analysis was performed in Matrix Science website, and all information related to target protein is highlighted. (PDF) [file pone.0142121.s002.pdf]

MATRIX SCIENCE MASCOT Search Results

**User**	: zhumuzi
**E-mail**	: zhumuzi@foxmail.com
**Search title**	: SCUT-150929
**Database**	: NCBInr 20150924 (71,615,283 sequences; 26,073,090,439 residues)
**Taxonomy**	: Firmicutes (gram-positive bacteria) (10,005,269 sequences)
**Timestamp**	: 29 Sep 2015 at 07:47:18 GMT

Not what you expected? Try **the select summary**.

▼Search parameters

**Type of search**	: MS/MS Ion Search
**Enzyme**	: Trypsin
**Fixed modifications**	: **Carbamidomethyl (C)**
**Variable modifications**	: **Acetyl (N-term)**, **Oxidation (M)**
**Mass values**	: Monoisotopic
**Protein mass**	: Unrestricted
**Peptide mass tolerance**	: ± 50 ppm (# ^{13}C = 1)
**Fragment mass tolerance**	: ± 0.5 Da
**Max missed cleavages**	: 1
**Instrument type**	: ESI-TRAP
**Number of queries**	: 1,138

▼Score distribution

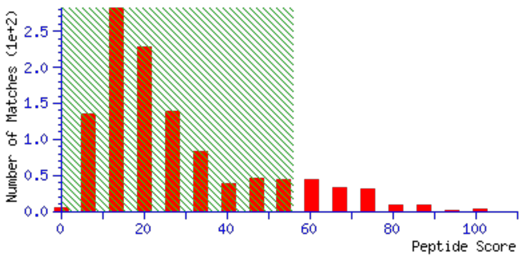

Peptide score distribution. Ions score is $-10\log(P)$, where P is the probability that the observed match is a random event.

There are **141** peptide matches above identity threshold and **263** matches above homology threshold for **1,138** queries. Histogram score range is (0, 101). On average, individual ions scores **> 56** (beyond green shading) indicate **identity or extensive homology** ($p<0.05$).

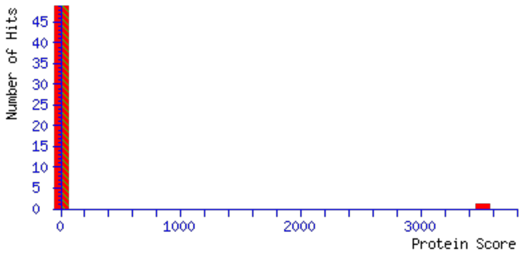

[Deprecated] Protein score distribution. Score distribution for family members in the first 50 proteins. Protein scores are derived from ions scores as a non-probabilistic basis for ranking protein families.

▼Modification statistics

**Modification**	**Site**	**Above thr.**
Oxidation	M	102
Carbamidomethyl	C	6

▼Legend

**Dupes**	**Expect**	**Rank**	**U**	**1**	**2**	**Peptide**	
	**0.037**	▶**2**				**GAYSLSLR**	significant
	9	▶1				GFFLFVEGGR	top ranking
	**6.4e-05**	▶**1**				**GSSIFGLAPGK**	significant and top ranking
	**1.3e-06**	▶**1**		■		**SSGTSYPDVLK**	peptide is found in all proteins in family member 1
	**6.2e-07**	▶**1**			■	**VCNYVSWIK**	peptide is found in some but not all proteins in family member 2
	**6.4e-05**	▶**1**	**U**			**GSSIFGLAPGK**	unique
▶**2**	**5.7e-05**	▶**1**				**LNTLETEEWFFK**	peptide has two duplicates
	0.18	▶*1*				*LNTLETEEWFFK*	duplicate peptide

Right-facing triangle (▶) in the Dupes or Rank column indicates content that can be expanded by clicking on it. Down-facing triangle (▼) indicates the content is expanded and can be collapsed. For more details about particular columns, see **results format help**.

Protein Family Summary

Significance threshold p< [0.05] Max. number of families [AUTO]

Ions score or expect cut-off 56 Dendrograms cut at 0

Preferred taxonomy All entries

Protein hits (4 proteins)

Filters: (none)

Export as CSV

↑Family	M	DB	Accession	Score	Mass	Matches	Match(sig)	Sequences	Seq(sig)	emPAI	Description
1	1	NCBInr	gi|504569872	3518	37235	142	138	18	18	16.29	catabolite control protein A [Thermoanaero
2	1	NCBInr	gi|916995283	64	39141	1	1	1	1	0.13	hypothetical protein [Ruminococcaceae ba
3	1	NCBInr	gi|497693278	63	36350	2	2	1	1	0.14	transcription regulator [Leuconostoc fallax]
4	1	NCBInr	gi|497670480	59	67686	1	1	1	1	0.07	aspartyl-tRNA synthetase [Ruminococcus f

Export as CSV

Not what you expected? Try **the select summary**.

Mascot: http://www.matrixscience.com/

MATRIX SCIENCE MASCOT Search Results

Protein View: gi|504569872

catabolite control protein A [Thermoanaerobacterium aotearoense]

**Database:**	NCBInr
**Score:**	3518
**Nominal mass (M_r):**	37235
**Calculated pI:**	5.69
**Taxonomy:**	Thermoanaerobacterium aotearoense

This protein sequence matches the following other entries:

- gi|389568645 from Thermoanaerobacterium saccharolyticum JW/SL-YS485
- gi|528281470 from Thermoanaerobacterium aotearoense
- gi|569525703 from Thermoanaerobacterium aotearoense SCUT27

Sequence similarity is available as an NCBI BLAST search of gi|504569872 against nr.

Search parameters

**Enzyme:**	Trypsin: cuts C-term side of KR unless next residue is P.
**Fixed modifications:**	Carbamidomethyl (C)
**Variable modifications:**	Acetyl (N-term), Oxidation (M)

Protein sequence coverage: 56%

Matched peptides shown in ***bold red***.

```
  1 MNATIKDVAR EAKVSIATVS RVLNNSAVVT DETKQRVLDA IKKTGYKPNA
 51 LARSLKIQKT HTIGLIVPDI SSPFYPEVVR GIEDIASMYS YNIFLCNTDQ
101 KEEKEMNYIE ILSEKQVDGI IYMGDIIRDS VKQQLKDIGI PIVLAGTEDA
151 ESEFPNVNID NKKASYDAVK YLISLGHKKI GMISGPADDP IGGVQRTNGY
201 KEALSEAKIR FKPSLVVEGS FKARQAYLAM LKLLENNVDA VFAASDEMAA
251 AAINAIFDSG FSVPEDIHVI GFDNTYLSYM FRPTITTIQR PAYDIGAVSM
301 RLMTKLLAKE PIDDMHVVLP HQLIVRESTG YGEEKK
```

Unformatted sequence string: 336 residues (for pasting into other applications).

Sort peptides by ◉ Residue Number ○ Increasing Mass ○ Decreasing Mass

Show predicted peptides also

Query	Start – End	Observed	Mr(expt)	Mr(calc)	ppm	M	Score	Expect	Rank	U	Peptide
260	1 – 10	559.8227	1117.6308	1117.5914	35.3	1	60	0.038	1	U	-.MNATIKDVAR.E
272	1 – 10	567.8209	1133.6272	1133.5863	36.1	1	68	0.0066	1	U	-.MNATIKDVAR.E + Oxidation (M)
273	1 – 10	567.8209	1133.6273	1133.5863	36.2	1	77	0.00083	1	U	-.MNATIKDVAR.E + Oxidation (M)
274	1 – 10	568.3118	1134.6090	1133.5863	902	1	56	0.11	2	U	-.MNATIKDVAR.E + Oxidation (M)
275	1 – 10	568.3131	1134.6117	1133.5863	905	1	58	0.063	2	U	-.MNATIKDVAR.E + Oxidation (M)
10	14 – 21	416.7615	831.5085	831.4814	32.6	0	60	0.017	1	U	K.VSIATVSR.V
11	14 – 21	416.7619	831.5092	831.4814	33.5	0	58	0.026	1	U	K.VSIATVSR.V
12	14 – 21	416.7622	831.5099	831.4814	34.3	0	60	0.017	1	U	K.VSIATVSR.V
14	14 – 21	416.7624	831.5103	831.4814	34.7	0	60	0.016	1	U	K.VSIATVSR.V
15	14 – 21	416.7624	831.5103	831.4814	34.8	0	57	0.036	1	U	K.VSIATVSR.V
16	14 – 21	416.7624	831.5103	831.4814	34.8	0	64	0.0062	1	U	K.VSIATVSR.V
17	14 – 21	416.7625	831.5104	831.4814	34.9	0	76	0.00044	1	U	K.VSIATVSR.V
18	14 – 21	416.7626	831.5107	831.4814	35.2	0	62	0.011	1	U	K.VSIATVSR.V
20	14 – 21	416.7627	831.5109	831.4814	35.4	0	67	0.0034	1	U	K.VSIATVSR.V
21	14 – 21	416.7628	831.5111	831.4814	35.7	0	60	0.017	1	U	K.VSIATVSR.V
22	14 – 21	416.7630	831.5114	831.4814	36.0	0	59	0.024	1	U	K.VSIATVSR.V
562	22 – 34	695.3871	1388.7596	1388.7147	32.3	0	74	0.00015	1	U	R.VLNNSAVVTDETK.Q
563	22 – 34	695.3871	1388.7597	1388.7147	32.4	0	87	2.9e-05	1	U	R.VLNNSAVVTDETK.Q
564	22 – 34	695.3872	1388.7599	1388.7147	32.5	0	74	5.1e-05	1	U	R.VLNNSAVVTDETK.Q
565	22 – 34	695.3874	1388.7603	1388.7147	32.8	0	73	5.6e-05	1	U	R.VLNNSAVVTDETK.Q
566	22 – 34	695.3875	1388.7605	1388.7147	32.9	0	87	2.8e-05	1	U	R.VLNNSAVVTDETK.Q
567	22 – 34	695.3876	1388.7607	1388.7147	33.1	0	76	6.3e-05	1	U	R.VLNNSAVVTDETK.Q
568	22 – 34	695.3876	1388.7607	1388.7147	33.1	0	87	8.9e-06	1	U	R.VLNNSAVVTDETK.Q
569	22 – 34	695.3877	1388.7608	1388.7147	33.2	0	68	8e-05	1	U	R.VLNNSAVVTDETK.Q
570	22 – 34	695.3877	1388.7608	1388.7147	33.2	0	68	7.8e-05	1	U	R.VLNNSAVVTDETK.Q
571	22 – 34	695.3877	1388.7609	1388.7147	33.3	0	73	0.00096	1	U	R.VLNNSAVVTDETK.Q
572	22 – 34	695.3877	1388.7609	1388.7147	33.3	0	73	5.4e-05	1	U	R.VLNNSAVVTDETK.Q
573	22 – 34	695.3877	1388.7609	1388.7147	33.3	0	74	5.4e-05	1	U	R.VLNNSAVVTDETK.Q
574	22 – 34	695.3881	1388.7617	1388.7147	33.9	0	74	6.9e-05	1	U	R.VLNNSAVVTDETK.Q
575	22 – 34	695.3889	1388.7631	1388.7147	34.9	0	87	8.7e-06	1	U	R.VLNNSAVVTDETK.Q
576	22 – 34	695.3891	1388.7636	1388.7147	35.2	0	74	6.4e-05	1	U	R.VLNNSAVVTDETK.Q
577	22 – 34	695.3892	1388.7638	1388.7147	35.3	0	87	2e-06	1	U	R.VLNNSAVVTDETK.Q
578	22 – 34	695.3892	1388.7639	1388.7147	35.4	0	74	5.2e-05	1	U	R.VLNNSAVVTDETK.Q
666	22 – 36	837.4748	1672.9351	1672.8744	36.3	1	88	3.7e-05	1	U	R.VLNNSAVVTDETKQR.V
236	44 – 53	545.8217	1089.6289	1089.5931	32.9	0	60	0.00059	1	U	K.TGYKPNALAR.S

244	44 - 53	545.8222	1089.6299	1089.5931	33.8	0	56	0.0018	1	U	K.TGYKPNALAR.S
938	60 - 80	1170.6703	2339.3260	2339.2525	31.4	0	98	6.4e-08	1	U	K.THTIGLIVPDISSPFYPEVVR.G
939	60 - 80	1170.6707	2339.3267	2339.2525	31.7	0	64	2.8e-05	1	U	K.THTIGLIVPDISSPFYPEVVR.G
941	60 - 80	1170.6715	2339.3285	2339.2525	32.5	0	66	1.4e-05	1	U	K.THTIGLIVPDISSPFYPEVVR.G
944	60 - 80	1170.6719	2339.3292	2339.2525	32.8	0	75	9.9e-07	1	U	K.THTIGLIVPDISSPFYPEVVR.G
945	60 - 80	1170.6719	2339.3292	2339.2525	32.8	0	71	1.9e-06	1	U	K.THTIGLIVPDISSPFYPEVVR.G
947	60 - 80	1170.6720	2339.3294	2339.2525	32.9	0	81	1.6e-06	1	U	K.THTIGLIVPDISSPFYPEVVR.G
948	60 - 80	1170.6721	2339.3297	2339.2525	33.0	0	61	2.9e-05	1	U	K.THTIGLIVPDISSPFYPEVVR.G
951	60 - 80	1170.6731	2339.3316	2339.2525	33.8	0	60	3.9e-05	1	U	K.THTIGLIVPDISSPFYPEVVR.G
956	60 - 80	1170.6738	2339.3331	2339.2525	34.4	0	61	4.3e-05	1	U	K.THTIGLIVPDISSPFYPEVVR.G
960	60 - 80	1170.6746	2339.3346	2339.2525	35.1	0	61	2.8e-05	1	U	K.THTIGLIVPDISSPFYPEVVR.G
961	60 - 80	1170.6752	2339.3358	2339.2525	35.6	0	58	0.00047	1	U	K.THTIGLIVPDISSPFYPEVVR.G
749	102 - 115	885.9561	1769.8975	1769.8393	32.9	1	59	0.0013	1	U	K.EEKEMNYIEILSEK.Q + Oxidation (M)
530	105 - 115	684.8615	1367.7085	1367.6642	32.3	0	67	0.0066	1	U	K.EMNYIEILSEK.Q
531	105 - 115	684.8619	1367.7092	1367.6642	32.9	0	61	0.027	1	U	K.EMNYIEILSEK.Q
532	105 - 115	684.8619	1367.7093	1367.6642	32.9	0	62	0.021	1	U	K.EMNYIEILSEK.Q
533	105 - 115	684.8621	1367.7096	1367.6642	33.2	0	65	0.012	1	U	K.EMNYIEILSEK.Q
536	105 - 115	684.8636	1367.7127	1367.6642	35.5	0	64	0.015	1	U	K.EMNYIEILSEK.Q
552	105 - 115	692.8597	1383.7048	1383.6591	33.0	0	56	0.084	1	U	K.EMNYIEILSEK.Q + Oxidation (M)
553	105 - 115	692.8598	1383.7050	1383.6591	33.2	0	68	0.006	1	U	K.EMNYIEILSEK.Q + Oxidation (M)
554	105 - 115	692.8599	1383.7052	1383.6591	33.3	0	64	0.014	1	U	K.EMNYIEILSEK.Q + Oxidation (M)
555	105 - 115	692.8599	1383.7053	1383.6591	33.4	0	74	0.0015	1	U	K.EMNYIEILSEK.Q + Oxidation (M)
556	105 - 115	692.8604	1383.7063	1383.6591	34.1	0	74	0.0016	1	U	K.EMNYIEILSEK.Q + Oxidation (M)
557	105 - 115	692.8606	1383.7067	1383.6591	34.4	0	57	0.07	1	U	K.EMNYIEILSEK.Q + Oxidation (M)
558	105 - 115	692.8607	1383.7069	1383.6591	34.5	0	71	0.0031	1	U	K.EMNYIEILSEK.Q + Oxidation (M)
559	105 - 115	692.8607	1383.7069	1383.6591	34.5	0	77	0.0007	1	U	K.EMNYIEILSEK.Q + Oxidation (M)
560	105 - 115	692.8611	1383.7076	1383.6591	35.0	0	73	0.0017	1	U	K.EMNYIEILSEK.Q + Oxidation (M)
561	105 - 115	692.8612	1383.7078	1383.6591	35.2	0	59	0.043	1	U	K.EMNYIEILSEK.Q + Oxidation (M)
618	116 - 128	746.9192	1491.8239	1491.7755	32.4	0	60	0.0012	1	U	K.QVDGIIYMGDIIR.D
619	116 - 128	746.9194	1491.8243	1491.7755	32.7	0	66	0.0014	1	U	K.QVDGIIYMGDIIR.D
620	116 - 128	746.9195	1491.8244	1491.7755	32.8	0	84	3.7e-06	1	U	K.QVDGIIYMGDIIR.D
621	116 - 128	746.9195	1491.8245	1491.7755	32.9	0	70	0.00016	1	U	K.QVDGIIYMGDIIR.D
622	116 - 128	746.9199	1491.8253	1491.7755	33.3	0	78	9.6e-05	1	U	K.QVDGIIYMGDIIR.D
623	116 - 128	746.9199	1491.8253	1491.7755	33.3	0	66	6.6e-05	1	U	K.QVDGIIYMGDIIR.D
624	116 - 128	746.9203	1491.8260	1491.7755	33.8	0	60	0.011	1	U	K.QVDGIIYMGDIIR.D
625	116 - 128	746.9205	1491.8264	1491.7755	34.1	0	65	0.0023	1	U	K.QVDGIIYMGDIIR.D
626	116 - 128	746.9205	1491.8264	1491.7755	34.1	0	58	0.027	1	U	K.QVDGIIYMGDIIR.D
627	116 - 128	746.9206	1491.8266	1491.7755	34.2	0	65	0.00058	1	U	K.QVDGIIYMGDIIR.D
628	116 - 128	746.9208	1491.8271	1491.7755	34.6	0	56	0.0034	1	U	K.QVDGIIYMGDIIR.D
635	116 - 128	754.9163	1507.8181	1507.7705	31.6	0	57	0.00063	1	U	K.QVDGIIYMGDIIR.D + Oxidation (M)
636	116 - 128	754.9170	1507.8194	1507.7705	32.5	0	66	0.002	1	U	K.QVDGIIYMGDIIR.D + Oxidation (M)
637	116 - 128	754.9171	1507.8197	1507.7705	32.6	0	62	0.0012	1	U	K.QVDGIIYMGDIIR.D + Oxidation (M)
638	116 - 128	754.9173	1507.8200	1507.7705	32.9	0	71	0.00012	1	U	K.QVDGIIYMGDIIR.D + Oxidation (M)
639	116 - 128	754.9174	1507.8202	1507.7705	33.0	0	69	0.00025	1	U	K.QVDGIIYMGDIIR.D + Oxidation (M)
640	116 - 128	754.9175	1507.8205	1507.7705	33.2	0	75	6.6e-05	1	U	K.QVDGIIYMGDIIR.D + Oxidation (M)
641	116 - 128	754.9176	1507.8206	1507.7705	33.2	0	73	0.00012	1	U	K.QVDGIIYMGDIIR.D + Oxidation (M)
642	116 - 128	754.9177	1507.8208	1507.7705	33.4	0	75	0.00046	1	U	K.QVDGIIYMGDIIR.D + Oxidation (M)
646	116 - 128	754.9188	1507.8231	1507.7705	34.9	0	75	0.00091	1	U	K.QVDGIIYMGDIIR.D + Oxidation (M)
647	116 - 128	754.9189	1507.8233	1507.7705	35.1	0	73	0.0001	1	U	K.QVDGIIYMGDIIR.D + Oxidation (M)
649	116 - 128	754.9191	1507.8237	1507.7705	35.3	0	74	4.6e-05	1	U	K.QVDGIIYMGDIIR.D + Oxidation (M)
650	116 - 128	754.9191	1507.8237	1507.7705	35.3	0	71	0.0021	1	U	K.QVDGIIYMGDIIR.D + Oxidation (M)
1032	137 - 162	924.1616	2769.4630	2769.3708	33.3	0	62	4.2e-05	1	U	K.DIGIPIVLAGTEDAESEFPNVNIDNK.K
1070	137 - 163	1449.7849	2897.5553	2897.4658	30.9	1	65	8.5e-06	1	U	K.DIGIPIVLAGTEDAESEFPNVNIDNKK.A
1071	137 - 163	1449.7851	2897.5557	2897.4658	31.0	1	58	1.4e-05	1	U	K.DIGIPIVLAGTEDAESEFPNVNIDNKK.A
1072	137 - 163	1449.7855	2897.5565	2897.4658	31.3	1	64	1.2e-05	1	U	K.DIGIPIVLAGTEDAESEFPNVNIDNKK.A
1073	137 - 163	1449.7864	2897.5582	2897.4658	31.9	1	57	0.00011	1	U	K.DIGIPIVLAGTEDAESEFPNVNIDNKK.A
1076	137 - 163	1449.7870	2897.5594	2897.4658	32.3	1	66	1.5e-05	1	U	K.DIGIPIVLAGTEDAESEFPNVNIDNKK.A
1077	137 - 163	966.8606	2897.5601	2897.4658	32.6	1	58	3.7e-05	1	U	K.DIGIPIVLAGTEDAESEFPNVNIDNKK.A
1078	137 - 163	966.8608	2897.5605	2897.4658	32.7	1	63	1.2e-05	1	U	K.DIGIPIVLAGTEDAESEFPNVNIDNKK.A
1079	137 - 163	966.8610	2897.5610	2897.4658	32.9	1	62	1.5e-05	1	U	K.DIGIPIVLAGTEDAESEFPNVNIDNKK.A
1080	137 - 163	966.8611	2897.5616	2897.4658	33.1	1	73	2.5e-06	1	U	K.DIGIPIVLAGTEDAESEFPNVNIDNKK.A
1082	137 - 163	966.8615	2897.5627	2897.4658	33.4	1	57	7.5e-05	1	U	K.DIGIPIVLAGTEDAESEFPNVNIDNKK.A
1085	137 - 163	1449.7892	2897.5638	2897.4658	33.8	1	61	1.8e-05	1	U	K.DIGIPIVLAGTEDAESEFPNVNIDNKK.A
1087	137 - 163	966.8622	2897.5649	2897.4658	34.2	1	62	2.3e-05	1	U	K.DIGIPIVLAGTEDAESEFPNVNIDNKK.A
1090	137 - 163	1449.7905	2897.5665	2897.4658	34.8	1	63	6.1e-06	1	U	K.DIGIPIVLAGTEDAESEFPNVNIDNKK.A
1091	137 - 163	966.8628	2897.5665	2897.4658	34.8	1	66	7.4e-05	1	U	K.DIGIPIVLAGTEDAESEFPNVNIDNKK.A
1092	137 - 163	966.8630	2897.5671	2897.4658	35.0	1	57	5e-05	1	U	K.DIGIPIVLAGTEDAESEFPNVNIDNKK.A
1097	137 - 163	1449.7937	2897.5728	2897.4658	36.9	1	58	8.5e-05	1	U	K.DIGIPIVLAGTEDAESEFPNVNIDNKK.A
768	179 - 196	914.0042	1825.9939	1825.9356	31.9	1	62	0.00012	1	U	K.KIGMISGPADDPIGGVQR.T + Oxidation (M)
667	180 - 196	841.9576	1681.9007	1681.8458	32.7	0	67	0.00013	1	U	K.IGMISGPADDPIGGVQR.T
668	180 - 196	841.9577	1681.9008	1681.8458	32.8	0	66	8e-05	1	U	K.IGMISGPADDPIGGVQR.T
669	180 - 196	841.9578	1681.9011	1681.8458	32.9	0	77	1.7e-05	1	U	K.IGMISGPADDPIGGVQR.T

670	180 - 196	841.9579	1681.9013	1681.8458	33.0	0	66	0.00022	1	U	K.IGMISGPADDPIGGVQR.T
672	180 - 196	841.9584	1681.9022	1681.8458	33.5	0	68	5.9e-05	1	U	K.IGMISGPADDPIGGVQR.T
675	180 - 196	841.9591	1681.9036	1681.8458	34.4	0	67	4.6e-05	1	U	K.IGMISGPADDPIGGVQR.T
676	180 - 196	841.9594	1681.9043	1681.8458	34.8	0	57	0.0007	1	U	K.IGMISGPADDPIGGVQR.T
677	180 - 196	841.9597	1681.9047	1681.8458	35.1	0	58	0.0011	1	U	K.IGMISGPADDPIGGVQR.T
679	180 - 196	841.9601	1681.9056	1681.8458	35.6	0	59	0.00078	1	U	K.IGMISGPADDPIGGVQR.T
680	180 - 196	841.9602	1681.9058	1681.8458	35.7	0	57	0.0012	1	U	K.IGMISGPADDPIGGVQR.T
686	180 - 196	849.9538	1697.8930	1697.8407	30.8	0	67	0.00015	1	U	K.IGMISGPADDPIGGVQR.T + Oxidation (M)
687	180 - 196	849.9548	1697.8950	1697.8407	32.0	0	84	2.3e-06	1	U	K.IGMISGPADDPIGGVQR.T + Oxidation (M)
689	180 - 196	849.9550	1697.8955	1697.8407	32.3	0	83	5.8e-06	1	U	K.IGMISGPADDPIGGVQR.T + Oxidation (M)
690	180 - 196	849.9551	1697.8957	1697.8407	32.4	0	75	4.5e-05	1	U	K.IGMISGPADDPIGGVQR.T + Oxidation (M)
691	180 - 196	566.9726	1697.8959	1697.8407	32.5	0	57	0.00092	1	U	K.IGMISGPADDPIGGVQR.T + Oxidation (M)
692	180 - 196	849.9553	1697.8961	1697.8407	32.6	0	83	4.7e-06	1	U	K.IGMISGPADDPIGGVQR.T + Oxidation (M)
693	180 - 196	849.9553	1697.8961	1697.8407	32.6	0	75	4.3e-05	1	U	K.IGMISGPADDPIGGVQR.T + Oxidation (M)
696	180 - 196	849.9554	1697.8963	1697.8407	32.8	0	89	1.1e-06	1	U	K.IGMISGPADDPIGGVQR.T + Oxidation (M)
698	180 - 196	849.9555	1697.8964	1697.8407	32.8	0	86	1.4e-06	1	U	K.IGMISGPADDPIGGVQR.T + Oxidation (M)
699	180 - 196	849.9555	1697.8964	1697.8407	32.8	0	82	3.1e-06	1	U	K.IGMISGPADDPIGGVQR.T + Oxidation (M)
700	180 - 196	849.9556	1697.8966	1697.8407	32.9	0	83	2.8e-06	1	U	K.IGMISGPADDPIGGVQR.T + Oxidation (M)
701	180 - 196	849.9557	1697.8968	1697.8407	33.1	0	101	5.2e-08	1	U	K.IGMISGPADDPIGGVQR.T + Oxidation (M)
702	180 - 196	849.9558	1697.8971	1697.8407	33.2	0	84	3.1e-06	1	U	K.IGMISGPADDPIGGVQR.T + Oxidation (M)
706	180 - 196	849.9570	1697.8995	1697.8407	34.6	0	79	1.1e-05	1	U	K.IGMISGPADDPIGGVQR.T + Oxidation (M)
707	180 - 196	849.9572	1697.8999	1697.8407	34.9	0	98	1e-07	1	U	K.IGMISGPADDPIGGVQR.T + Oxidation (M)
708	180 - 196	849.9573	1697.9000	1697.8407	34.9	0	87	1.4e-06	1	U	K.IGMISGPADDPIGGVQR.T + Oxidation (M)
710	180 - 196	849.9574	1697.9002	1697.8407	35.1	0	63	0.00017	1	U	K.IGMISGPADDPIGGVQR.T + Oxidation (M)
492	197 - 208	656.3467	1310.6788	1309.6514	785	1	63	0.02	1	U	R.TNGYKEALSEAK.I
495	197 - 208	656.3487	1310.6828	1309.6514	788	1	76	0.00016	1	U	R.TNGYKEALSEAK.I
497	197 - 208	656.3574	1310.7002	1309.6514	801	1	67	0.004	1	U	R.TNGYKEALSEAK.I
502	211 - 222	669.3989	1336.7832	1336.7391	33.0	0	59	0.01	1	U	R.FKPSLVVEGSFK.A
509	211 - 222	669.3998	1336.7851	1336.7391	34.4	0	69	0.0015	1	U	R.FKPSLVVEGSFK.A
516	211 - 222	669.4006	1336.7866	1336.7391	35.5	0	59	0.0028	1	U	R.FKPSLVVEGSFK.A
989	306 - 326	818.1567	2451.4484	2451.3672	33.1	1	58	8.9e-05	1	U	K.LLAKEPIDDMHVVLPHQLIVR.E + Oxidation (M)
827	310 - 326	1014.0734	2026.1322	2026.0670	32.2	0	60	3e-05	1	U	K.EPIDDMHVVLPHQLIVR.E + Oxidation (M)
835	310 - 326	1014.0742	2026.1338	2026.0670	33.0	0	71	1.1e-05	1	U	K.EPIDDMHVVLPHQLIVR.E + Oxidation (M)
841	310 - 326	1014.0748	2026.1351	2026.0670	33.6	0	60	0.00032	1	U	K.EPIDDMHVVLPHQLIVR.E + Oxidation (M)
166	327 - 335	500.2350	998.4555	998.4193	36.3	0	57	0.013	1	U	R.ESTGYGEEK.K

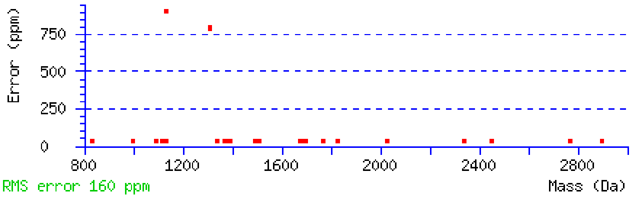


```
LOCUS       WP_014756974             336 aa            linear   BCT 26-JUN-2015
DEFINITION  catabolite control protein A [Thermoanaerobacterium aotearoense].
ACCESSION   WP_014756974
VERSION     WP_014756974.1  GI:504569872
KEYWORDS    RefSeq.
SOURCE      Thermoanaerobacterium aotearoense
  ORGANISM  Thermoanaerobacterium aotearoense
            Bacteria; Firmicutes; Clostridia; Thermoanaerobacterales;
            Thermoanaerobacterales Family III. Incertae Sedis;
            Thermoanaerobacterium.
COMMENT     REFSEQ: This record represents a single, non-redundant, protein
            sequence which may be annotated on many different RefSeq genomes
            from the same, or different, species.
            COMPLETENESS: full length.
FEATURES             Location/Qualifiers
     source          1..336
                     /organism="Thermoanaerobacterium aotearoense"
                     /db_xref="taxon:47490"
     Protein         1..336
                     /product="catabolite control protein A"
                     /calculated_mol_wt=37071
```

```
     Region          3..330
                     /region_name="PurR"
                     /note="DNA-binding transcriptional regulator, LacI/PurR
                     family [Transcription]; COG1609"
                     /db_xref="CDD:224525"
     Region          6..56
                     /region_name="HTH_LacI"
                     /note="Helix-turn-helix (HTH) DNA binding domain of the
                     LacI family of transcriptional regulators; cd01392"
                     /db_xref="CDD:143331"
     Site            order(6,14..18,20..21,24,29..30,33,46,49,52..53,55..56)
                     /site_type="DNA binding"
                     /note="DNA binding site [nucleotide binding]"
                     /db_xref="CDD:143331"
     Site            46..55
                     /site_type="other"
                     /note="domain linker motif"
                     /db_xref="CDD:143331"
     Region          62..328
                     /region_name="Periplasmic_Binding_Protein_Type_1"
                     /note="Type 1 periplasmic binding fold superfamily;
                     cl10011"
                     /db_xref="CDD:277459"
```

Mascot: http://www.matrixscience.com/
